# An Empirically Based, Recovery‐Orientated Framework for Safety Planning Interventions for Suicide Prevention: An Integrative Literature Review and Critical Meta‐Autoethnographic Synthesis

**DOI:** 10.1111/jpm.70202

**Published:** 2026-09-09

**Authors:** Amanda Kate Green, Alan Simpson, Vasiliki Tzouvara, Corinna Hackmann, Eva Klotz, Mary Leamy

**Affiliations:** ^1^ Florence Nightingale Faculty of Nursing, Midwifery & Palliative Care King's College London London UK; ^2^ Norfolk & Suffolk NHS Foundation Trust Norwich Norfolk UK; ^3^ Charité University Medicine Berlin Berlin Germany

**Keywords:** autoethnography, lived experience, peer support, personal recovery, safety planning intervention, suicidal ideation, suicide prevention

## Abstract

**Introduction:**

Safety planning interventions are increasingly used in suicide prevention, yet their design and delivery often lack alignment with personal recovery principles.

**Aim:**

To explore with lived experience, empirical literature on safety planning for suicide prevention and examine the relationship between change mechanisms and personal recovery concepts.

**Method:**

An integrative literature review was conducted combined with critical meta‐autoethnographic synthesis. Data were analysed using the Donabedian Model of Healthcare, the Conceptual Framework for Personal Recovery, lived experience and translated using reciprocal and refutational synthesis.

**Results:**

Fifteen studies were included. An empirically based recovery‐orientated framework was developed comprising three phases of personal development—*Inspiring, Discovering* and *Embodying*—and three mechanisms of change—*Learning, Introspection* and *Self‐reflection*. The framework reconceptualises the clinicians' role as ‘facilitator’ highlighting the importance of relational, personalised approaches.

**Discussion:**

The findings challenge dominant biomedical approaches, offering a more personalised, recovery‐focused understanding of safety planning interventions.

**Implications for Practice:**

The framework supports delivery of person‐centred care, policy implementation, service redesign and workforce training aligned with NHS and WHO guidance.

**Recommendations:**

Future research should evaluate the framework's effectiveness and explore its application in diverse populations and settings.

## Accessible Summary

- What is known about the subject?
  ○ Safety planning interventions (SPIs) are widely used to help people stay safe during suicidal crises.
  ○ Most existing SPIs are brief, clinician‐led and focus mainly on reducing risk and improving clinical outcomes.
  ○ They often lack personalisation and do not fully reflect the experiences or needs of people with severe mental illness.
- What does this paper add to existing knowledge?
  ○ It introduces a new recovery‐orientated framework for SPIs, developed through a review of literature and reflexive use of personal lived experience.
  ○ The framework includes three phases of personal development—Inspiring, Discovering and Embodying—and three mechanisms of change—Learning, Introspection and Self‐reflection.
  ○ It uses an innovative method called critical meta‐autoethnographic synthesis, combining integrative literature review with lived experience to create new insights.
  ○ It reframes the clinician's role from risk manager to facilitator, promoting relational and personalised care.
- What are the implications for practice?
  ○ Mental health professionals and peer support workers can use the framework to co‐produce person‐centred, recovery‐focused safety planning interventions.
  ○ It supports flexible and adaptive care for people with complex mental health needs, including those with SMI.
  ○ The framework can guide service redesign, staff training and policy implementation in line with NHS and WHO suicide prevention guidance.

## 1 Introduction

Severe mental illness (SMI) is a major contributor to premature mortality worldwide, with suicide recognised as one of the leading causes of death (World Health Organization 2024). Suicidality, defined in this study as ‘suicidal thoughts, feelings and behaviours’, is inextricably linked with many SMIs. Certain major mood disorders, such as *bipolar disorder*, for example, increase suicide risk twenty‐fold (Kutcher and Chehil 2012). Other psychiatric conditions frequently implicated include *schizophrenia* and *personality disorders* (Pompili et al. 2005; Bertolote et al. 2004), *obsessive‐compulsive disorder* (Angelakis et al. 2015) and *post‐traumatic stress disorder* (Chang et al. 2011). Despite international mental health policy over the past two decades emphasising a commitment to a major paradigm shift towards a personal recovery‐orientation (Llewellyn‐Beardsley et al. 2019), people with SMIs are seeking treatment from psychiatric services which remain dominated by a biomedical approach. Suicide prevention practices of psychiatric patients which are focused on observation, coercion and clinician control (Kutcher and Chehil 2012) appear not to work for many patients, particularly within acute (inpatient) settings. This is highlighted by a report from the National Confidential Inquiry into Suicide and Safety in Mental Health which reports that ‘28% of mental health patient deaths by suicide in the UK occurred whilst the patient was under enhanced nursing observation on the psychiatric ward’ (NCISH 2024, 22). The transformation of risk management within psychiatric services is an urgent priority, and approaches which foreground personal recovery could address a crucial quality gap. Shifting focus from clinical control to self‐management, personal responsibility and agency (Schrank et al. 2012) could confront issues of dependency, self‐stigma and helplessness among service users (Borg and Kristiansen 2009).

NHS England recently issued best practice guidance recommending a safety planning approach for assessing, formulating and managing suicide risk (NHS England 2025). The National Institute for Health and Care Excellence have also recommended the use of a ‘safety plan’ for reducing self‐harm and suicidality, (NICE 2022), yet there is a surprising lack of evidence for the efficacy of safety plans for people with severe mental illness (SMI) (House 2022). The original safety planning intervention (SPI) (Stanley and Brown 2012), derived from cognitive behavioural therapy (Stanley et al. 2009) is a brief intervention for veterans in America admitted to an emergency department following a suicide‐related event. The intervention is focused on creating a *safety plan* described as ‘a written prioritised list of coping strategies, resources and sources of support an individual should use when in a suicidal crisis’ (Stanley and Brown 2012, 258) and doing so in the shortest amount of a clinicians' time.

According to the Medical Research Council's (MRC) guidance, ‘an intervention might be considered “complex” because of properties of the intervention itself, such as the number of components involved; the range of behaviours targeted; expertise and skills required by those delivering and receiving the intervention; […] the permitted level of flexibility of the intervention or its components’ (Skivington et al. 2021, 2). Given this definition, it is reasonable to classify SPIs as complex interventions, due to the targeting of suicidality, as well as the high levels of flexibility permitted in the intervention structure and resources. This includes tailoring the intervention in person‐centred ways and flexibility in how, how often, where and by whom it is delivered. A preliminary literature search did not identify a review that was led by a lived experience researcher who synthesised the safety planning literature through the lens of lived experience. Our preliminary literature search did reveal there is no standardised definition of safety planning, descriptions of its major components, guidelines for practice or standard model (Logan and Walker 2018; Dworkin et al. 2016). In an attempt to address this, we conducted a systematic integrative literature review following established methods (Whittemore and Knafl 2005), combined with a critical meta‐autoethnographic synthesis, in order to incorporate a lived experience perspective (Beames et al. 2021). The rationale for adopting an autoethnographic approach is to build on the literature of SPIs to help interpret and develop a new, empirically based recovery‐orientated safety planning framework informed by lived experience. This study explores the nuances of safety planning by drawing on AG's personal experiences of suicidality and her work supporting others in peer support in secondary mental health care. She consciously uses these experiences and reflections to construct new interpretations and identify priorities of focus for SPIs.

The specific questions guiding this research are: (1) What SPIs have been developed and how do they work for adults experiencing suicidal ideation and/or self‐harming behaviours to increase physical and psychological safety? (2) What is the relationship between conceptual and/or theoretical understandings of how SPIs work and how personal recovery concepts and theoretical understandings work to increase safety behaviours and outcomes?

## 2 Method

We conducted a systematic integrative literature review (ILR) using the methodology proposed by Whittemore and Knafl (2005) in order to gain an in‐depth analysis of the published literature on SPIs. We combined this with critical meta‐autoethnographic synthesis which is an adaptation of meta‐ethnography (Noblit and Hare 1988). Meta‐ethnography allows the interpretive properties of primary data to be analysed and reviewers to produce new interpretations that go beyond the findings of individual studies (France et al. 2019). It is also useful for understanding the inter‐relationships across studies and considering the contextual and social factors underpinning theoretical frameworks and models (Bearman and Dawson 2013). This approach was adapted to use the autoethnographic understandings of the first author AG. Critical meta‐autoethnographic synthesis is an approach to the whole process of the review, rather than to just the synthesis component in that a lived experience approach was taken to the entire review and provides a critical lens to the literature in terms of deconstructing research traditions or theoretical assumptions as a means of contextualising findings. The rationale for combining ILR methodology with critical meta‐autoethnographic synthesis is to build on the literature of SPIs to help interpret and develop new conceptualisations, informed by lived experience knowledge, since ILRs allow for the inclusion of diverse methodologies to explore different perspectives on a topic (Toronto and Remington 2020). Lived experience in this review was used to relate to and interpret the data in order to develop an understanding of the underlying processes and the mechanisms of change for the person using safety planning. The review protocol was registered with PROSPERO (CRD42024533324). See Table 1 for critical meta‐autoethnographic synthesis method. (See File S1 for critical meta‐autoethnographic synthesis methodology).

**TABLE 1:** Critical meta‐autoethnographic synthesis method.

Phases	Description
*Phase 1*: Selecting primary & secondary methodologies (n.b. CMAS always secondary methodology)	Justification & Eligibility for both methodologies For example, Primary Methodology = Integrative Literature Review Secondary Methodology = Critical Meta‐Autoethnographic Synthesis
*Phase 2*: Conducting study using primary methodology	Searches Screening Reading included studies Appraisal Data extraction
*Phase 3*: Lived experience relevance to study	Deciding what part of own lived experience is relevant to study: scope, ethical considerations Criteria for: Lived experience as DATA Lived experience as interpretive lens
*Phase 4*: Determining how studies and lived experience are related	Considering and comparing study characteristics and lived experience Included studies data extraction for RQ1: TIDieR Checklist Donabedian Model Included studies data extracted for RQ2: Conceptual Framework for Personal Recovery Using lived experience as interpretive lens
*Phase 5*: Translating lived experience data and studies into one another	Using reciprocal and refutational translations
	Using lived experience data set
*Phase 6*: Synthesising translations	Lines‐of‐argument syntheses
	Using lived experience as interpretive lens
*Phase 7*: Expressing synthesis	Using lived experience as interpretive lens
	Define and describe empirically based, lived experience informed, recovery‐orientated safety planning framework

### 2.1 Literature Search Strategy

The search strategy was developed in collaboration with a university librarian. The PICO framework (Richardson et al. 1995) was used to develop search strategies and define eligibility criteria (see Table 2). The focus was on database searches in order to extract evidence from primary empirical qualitative, quantitative and mixed method studies.

**TABLE 2:** Databases and search strategy (PICO).

Databases searched (August 2024—October 2024)
CINAHL Embase Scopus MEDLINE PubMed

Key search terms
Safety plan(ning) OR crisis plan(ning) OR risk management plan(ning) AND suicide prevention OR self‐harm OR self‐injurious behaviour

Include	Exclude
*Population*	
People 16 years or older, any gender People living with suicidal ideation and/or behaviours (suicide attempt, non‐fatal, DSH) Formal carers (healthcare professionals, charity volunteers) Informal carers (friends, family members) Peer support workers Any mental health illness or disorder Any health care setting (mental health or general hospital) or community treatment Any non‐health care settings	People 15 years of age or younger People diagnosed with any organic mental disorder (e.g., dementia) People with brain injury
*Intervention/Exposure*	
Safety plan used contains as a minimum personalised coping strategies and sources of support	Studies where safety planning was not the central intervention being studied
Digital (e.g., mobile phone apps) or pen and paper formats	The safety plan used was a generic clinician generated list of coping strategies and ‘typical triggers’ to suicidal feelings
Virtual or in‐person delivery of intervention	Another suicide prevention intervention as primary intervention of study
Group or 1‐to‐1 delivered intervention	Safety planning to escape domestic violence
*Comparator/Context*	
Any comparator or control group (i.e., TAU, other suicide prevention intervention, clinician compared to peer support worker delivered) Self‐administered safety planning interventions	None
*Outcome*	
Feasibility and health system considerations Level of skill required by those delivering the intervention Level of skill required by those receiving the intervention Interaction of SPIs with context and adaptation Balance of health benefits and harms Multiple health and non‐health outcomes Personal recovery outcomes Personal change processes Human rights and sociocultural acceptability	Pathology and diagnosed symptoms (i.e., depression outcomes)
*Study characteristics*	
All study designs reporting on qualitative, quantitative or mixed method of an SPI Any date	Not published in English Protocol of study Literature Review

### 2.2 Data Extraction

A data extraction table captured the key study characteristics, namely lead author and date, country where the study was conducted, study design, setting, population, co‐production/lived experience in research.

### 2.3 Quality Appraisal

All studies included in this review underwent critical appraisal for quality using the Mixed Methods Appraisal Tool (MMAT) (Hong et al. 2018). MMAT is designed to critically appraise quantitative, qualitative and mixed methods studies included in systematic mixed‐studies reviews. Two reviewers (AG & EK) independently conducted a quality appraisal of the studies, rating studies on individual quality items and producing an overall score. Any discrepancies were resolved between them or by discussion with other authors. Whilst methodological quality was taken into consideration during analysis, no studies were excluded based on their quality to avoid possible exclusion of valuable findings (Whittemore and Knafl 2005).

### 2.4 Data Analysis

Two data analysis approaches were used to answer research question 1. Firstly, the Template for Describing and Replicating Interventions (TIDieR) (Hoffman et al. 2014) was completed by two authors (AG & EK) independently, which gave an overview of the key characteristics of the SPIs. Secondly, verbatim data from included studies was extracted using the Donabedian Model of Healthcare Quality (Donabedian 1988). To answer research question 2, verbatim data from included studies were extracted according to the Conceptual Framework for Personal Recovery in Mental Health (Leamy et al. 2011). See Table 3 for data extraction frameworks and key elements.

**TABLE 3:** Data extraction frameworks and key elements.

Research question	Framework/Model	Key elements
RQ1: What safety planning interventions have been developed and how do they work for adults experiencing suicidal ideation and/or self‐harming behaviours to increase physical and psychological safety?	TIDieR Checklist	*Structure*: Why, what‐ materials & procedures, who provided, how, where, when and how much, tailoring, modifications, how well planned/actual
	Donabedian Model	*Processes*: Intervention focus, decision‐making, theoretical knowledge application, social relations, self‐knowledge & awareness *Outcomes*: Changes related to knowledge, changes related to attitudes, changes related to behaviour, challenges related to structure, challenges related to processes
	Suicide‐related quantitative/qualitative data	*Outcomes*: ideation, behaviours, healthcare visits, satisfaction, usability. Effects on depression/suicidality/safety plan content, deliverer/user quotes
RQ2: What is the relationship between conceptual/theoretical understandings of SPIs and personal recovery concepts to increase safety behaviours and outcomes?	CHIME framework	*CHIME*: Connectedness, hope & optimism, identity, meaning & purpose, empowerment

### 2.5 Data Synthesis

We used a multifaceted approach to synthesis which we term here *critical meta‐autoethnographic synthesis* and is an adaptation of meta‐ethnographic synthesis in the following ways. Firstly, the intentional use of the lived experience of AG (autoethnography) to define the theories and conceptualisations that are produced from the integration or comparison of findings from included studies. Secondly, in the application of the approach to include data from mixed methods and quantitative studies. Analogous to meta‐analysis, meta‐synthesis is used to integrate multiple studies in order to produce comprehensive and interpretive findings; however, compared to meta‐analysis, which reduces quantitative studies into averages, meta‐synthesis enlarges findings by evaluating the uniqueness of an individual study into a comprehensive and interpretive whole (Clemmens 2003).

Integration of individual studies was achieved by using the meta‐ethnography approach suggested by Noblit and Hare (1988): reciprocal, refutational and line‐of‐argument. Reciprocal synthesis finds the harmony between studies and autoethnography, which can be combined based on the assumption that there is similarity between them. When this assumption of similarity is not valid, a refutational approach is used. Refutational synthesis treats the counterevidence itself as part of the competing explanations, and the implied refutation is incorporated into the synthesis. The lines‐of‐argument synthesis is essentially a process of inference and the construction of an interpretation at a higher level of abstraction (Noblit and Hare 1988), explaining how the different concepts and findings fit together to form a comprehensive argument.

For this review, five stages of synthesis and integration were performed:

*Stage 1*: Summary synthesis of data extraction tables—TIDieR checklist and Donabedian Model were produced that focused on answering RQ1.

*Stage 2*: Summary synthesis of results of CHIME recovery processes was produced that focused on answering RQ2.

*Stage 3*: Translation table developed by tabulating: column 1 = summary synthesis Stage 1; column 2 = summary synthesis Stage 2; column 3 = autoethnography/lived experience; column 4 = reciprocal comparisons between columns 1–3; column 5 = refutational contrasts between columns 1–3.

*Stage 4*: Translation table used to develop separate lines‐of‐argument syntheses (LOA) for: (i) intervention structure and resource; (ii) changes related to behaviour; (iii) changes related to attitudes, including external & internal factors; (iv) changes related to, and ongoing pursuit of, knowledge.

*Stage 5*: Lines‐of‐argument syntheses used to define and describe empirically based, lived experience informed, recovery‐orientated safety planning framework.

### 2.6 Ethical Considerations

This literature review was undertaken as part of a wider realist evaluation examining safety planning in mental health contexts. The realist evaluation has ethical approval IRAS293012, is being conducted in an NHS mental health trust and is sponsored by King's College London.

#### 2.6.1 Reflexivity Statement

An auto‐ethnographical approach required AG to draw on personal experience of using a safety planning intervention, a reflective process involving balancing personal reflection and rigorous analysis. To strengthen the rigour of this reflexive process, AG worked deliberately to avoid both false objectivity and unexamined bias, remaining constantly aware of their own biases and how these might be influencing interpretation. Reflexivity was embedded throughout as AG continually examined their role, interpretive lens and positionality, discussing these with co‐authors to support transparency and accountability. Methodological rigour achieved through the use of critical meta‐autoethnographic synthesis enabled transparency of the integration of lived experience with data from included studies.

Ethical safeguards were also applied to protect others who may appear in lived experience narratives, for example, involved mental health professionals, friends or family. To ensure privacy and respect for any individual, no other person is depicted or described in any way in this review. Consideration of the respect for participant privacy was also given and included studies were reviewed to ensure they had sufficient safeguards that respected subject confidentiality. Secondary data was handled to ensure the anonymity of the original participants.

*Caring for self* is a deliberate and ongoing practice, and decisions about what to share or omit were guided by long‐standing professional principles of relevance, purpose and meaning making in the intentional use of lived experience, learned through many years working in mental health peer support. AG also considered the ethical responsibility owed to readers, recognising that reflections on suicidality may evoke strong emotions and trusting readers to manage their own emotions in the way they deem most appropriate.

This integrated ethical stance, protecting self, safeguarding others and caring for readers, underpinned the use of lived experience through this study, ensuring that its inclusion minimised harm and maximised benefit by contributing to the overall analysis of the literature on SPIs.

## 3 Results

The results of this review are reported using the Preferred Reporting Items for Systematic Reviews and Data Analysis (Page et al. 2021). A visual summary of the process used to include and exclude studies is provided in the PRISMA flow diagram (Figure 1). Next, the results are reported according to the stages of the synthesis.

**FIGURE 1:**
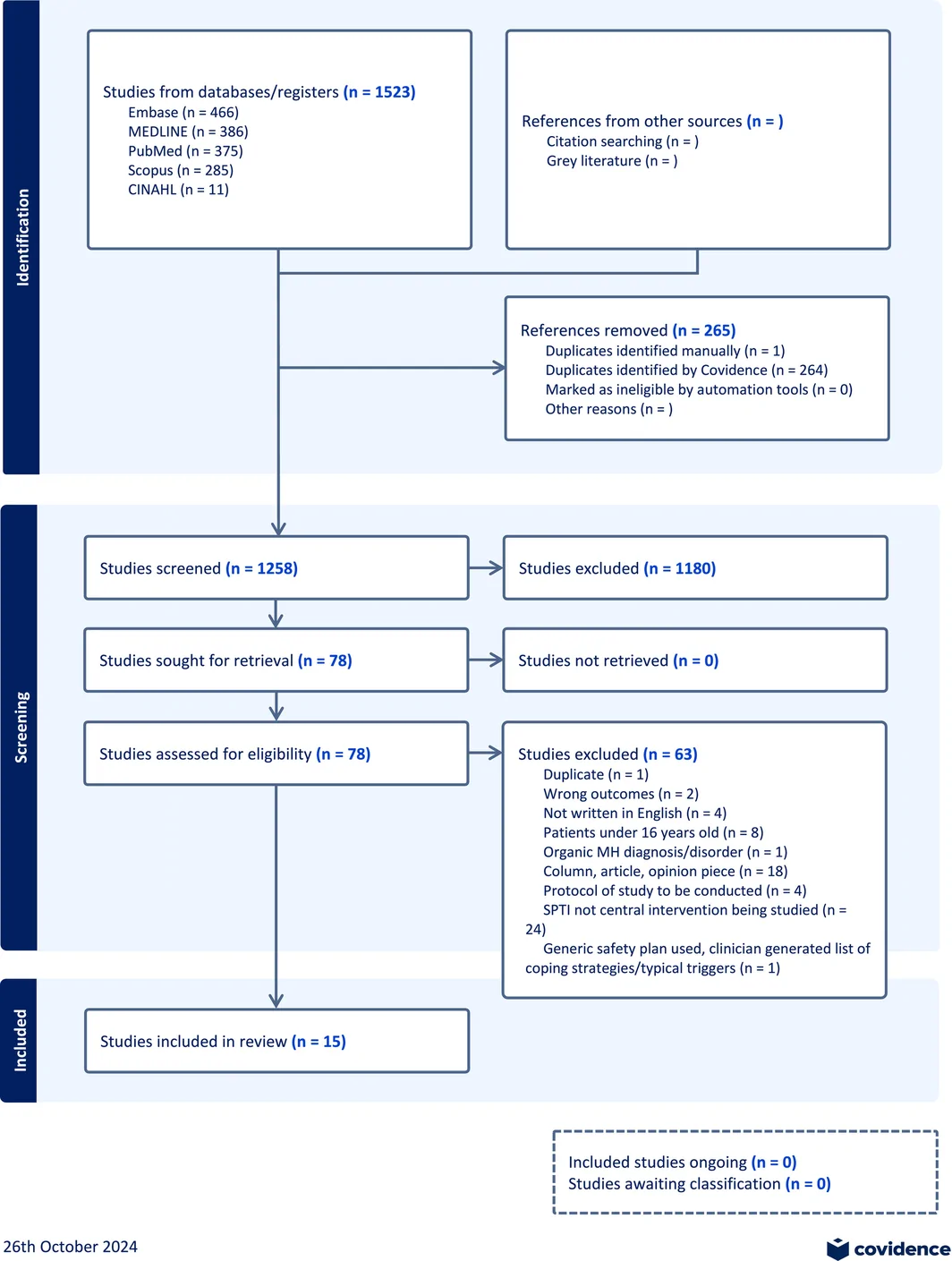
PRISMA flow diagram.

### 3.1 Searches

The literature search yielded 1523 studies. These were imported into Covidence Systematic Review Software (Covidence systematic review software, n.d.), which automatically removed 265 duplicate articles. The study titles and abstracts were screened for eligibility based on inclusion and exclusion criteria, leading to 1180 studies being excluded. The remaining full text of 78 studies was screened, leading to 63 studies being excluded and 15 studies included in the review. (See Figure 1).

### 3.2 Study Characteristics and Quality Appraisal

Results of study characteristics and quality appraisal are reported in Table 4.

**TABLE 4:** Study characteristics and quality appraisal.

Lead Author & Date	Country	Study design	Setting	Population/Participants	Co‐production/Lived experience in research	MMAT quality criteria (yes = 1, no = 0, cannot tell = 0).				
						1	2	3	4	5
Boudreaux et al. (2017)	United States	Quasi experimental Mixed Methods: Qualitative & Quantitative Descriptive	An urban, tertiary care hospital in Central Massachusetts.	30 Adult patients being evaluated for an acute psychiatric emergency in either the ED or inpatient psychiatric unit.	Co‐design of a web‐based, self‐administered safety planning application.	0	0	0	0	0
						1	0	0	0	0
						0	0	0	0	0
Chesin et al. (2015)	United States	Pilot Randomised Controlled Trial (Quantitative)	Psychotherapy outpatient clinic	18 individuals who endorsed current suicidal ideation and had a history of serious suicidal ideation (i.e., with method or plan) or a suicide attempt within the past 6 months. Participants were required to be engaged in individual mental health treatment (psychotherapy excluding DBT and ACT and/or psychiatric medication management)	None	1	0	0	0	0
						1	1	0	0	1
						0	0	0	0	0
Goodman et al. (2023)	United States	Pilot Randomised Controlled Trial (quantitative)	A large Veteran Affairs Medical Center in a major metropolitan areas in the Northeast	31 veteran‐supporting partner dyads struggling with recent suicidal ideation or past suicide behaviour	None	0	1	0	0	1
Goodman et al. (2020)	United States	Open Trial (Quantitative non‐randomised study)	James J. Peters VA medical center (Bronx, NY)	31 Veterans	None	0	1	0	0	0
Melvin et al. (2019)	Australia	Mixed methods (Qualitative and Quantitative non‐randomised study)	Tertiary mental health service	36 non‐aboriginal Australians aged between 16 and 42 years	None	1	1	0	1	0
						0	1	0	0	0
						0	0	0	0	0
O'Connor et al. (2022)	UK	Pilot Randomised Controlled Trial (quantitative)	NHS Liaison Psychiatry team clinicians based in four general hospitals in Scotland	120 patients admitted to one of four general hospitals following a self‐harm episode where there was evidence of suicidal intent	PPI group informing research team	1	1	1	1	1
						1	1	1	0	1
						1	1	1	0	1
Parrish et al. (2023)	United States	Quantitative randomised controlled trial (quantitative)	Multiple psychiatric walk‐in clinics and community mental health centers in San Diego County	53 people with SMI at risk of suicide	None	1	0	0	0	0
Patel et al. (2023)	United States	Qualitative	Two Veteran Affairs medical centers: James J Peters VA Medical Center in the Bronx, NY and Michael j. Crescenz VA medical center in Philadelphia, PA. To deliver PLF via telehealth, the research team utilised either VA video connect or VA WebEx software	17 Veterans	None	1	1	1	1	1
Porras‐Segovia et al. (2024)	Spain	Mixed Methods Qualitative and non‐randomised study (quantitative)	Digital safety plan embedded in the MEmind app which is free, compatible with iOS and Android	51 patients with a history of recent suicidal behaviour or suicidal ideation (in the month prior to recruitment) recruited in the emergency department, in medical units, in the mental health inpatient unit and in the mental health consultations of two hospitals in Madrid (Spain)	None	1	1	0	1	1
						0	0	0	0	0
						1	0	0	0	0
Rainbow, Tatnell, and Blashki (2024)	Australia	Non‐randomised study (Quantitative)	Website of Beyond Blue – a national mental health service and advocacy organisation in Australia	131 Visitors to the website of Beyond Blue who self‐reported having a current suicide safety plan and a lifetime history of suicidal thoughts and/or behaviours	Lived experience researcher on research team	1	1	1	0	0
Rainbow, Tatnell, Blashki, and Melvin (2024)	Australia	Mixed Methods Qualitative and quantitative descriptive study	Beyond Now mobile phone application freely available to download in Australia on both iOS and Android platforms. It was designed specifically to enable users to create a safety plan to support them through a suicide crisis via prioritised steps. These steps are based on the structure of the SPI (Stanley and Brown 2012) with language adaptations for Australian audiences	150 People residing in Australia and current users of the Beyond Now mobile phone app	Lived experience researcher on research team	1	1	1	1	1
						1	1	1	0	1
						1	1	0	0	1
Shah et al. (2024)	UK	Qualitative	Four emergency Departments in general hospitals in Southeast England	12 psychiatric liaison practitioners and 46 patients presenting to the emergency department with self‐harm and/or suicidal ideation	PPI group informing research team	1	1	1	1	1
Stanley et al. (2018)	United States	Non‐randomised study	Five Veteran Affairs ED intervention sites where SPI+ was implemented as standard care and four VA ED comparison usual care sites	1640 (intervention and comparison groups) veterans who came to the ED for a suicidal crisis and were determined not to require inpatient hospitalisation	None	0	1	1	1	0
Vijayakumar et al. (2017)	India	Randomised controlled trial (quantitative)	Two refugee camps in Southern India	265 Refugees (predominantly from Sri Lanka) residing in two refugee camps	None	0	0	0	0	0
Wilson et al. (2022)	United States	Randomised controlled trial	The Department of Emergency Medicine at the University of Arkansas for medical Sciences in Little Rock	15 clinicians and 16 peer support workers delivering the SPI	Peer support worker delivered intervention	1	1	0	0	0

### 3.3 Synthesis

The results have been reported following the stages of the synthesis:

#### 3.3.1 Stage 1

The completed TIDieR checklist and Donabedian Model results were summarised and tabulated—see Table 5. (See File S2 for completed TIDieR checklist. See File S3 for Stage 1 synthesis Donabedian Model).

**TABLE 5:** Stage 1 syntheses summary: Results TIDieR checklist & Donabedian Model.

Structure	
Donabedian Model categories	TIDieR
Engaging with intervention as useful resource Reliable & consistent delivery Flexible, simple, practical intervention format Collaborating to co‐author safety plan Relevance of people & activities (personalisation)	Safety planning interventions: Delivery: 1‐2‐1/group/Digital Self‐administered/clinician delivered Single session/multiple sessions Therapy techniques taught/no teaching within intervention
Venue Institutional culture as challenge Using technology for self‐destructive behaviour (digital SPIs)	Venue Hospital ER Department Psychiatric outpatient clinics Virtual/on‐line/telephone
Safety plan Forward & contingency planning Living document Remembering content Timing of accessing/referring to it Generic/multiple choice content Template headings	Safety plan Use of SP template with predetermined headings Multiple choice options/free text SP contains six steps of original SPI (7th as an addition) SP app/web‐site/pen & paper Contains generic content
Focus is on Solution‐focused approach Scope/intervention reach	SPI focus Increase time efficiency of building a safety plan Combine SPI with learning therapeutic techniques Involve others (professional & family) for support Provide additional support after discharge Increase suicide‐related coping
Intervention source (who delivers SPI) Therapeutic relationship: Guided reflection enabling introspection & self‐reflection Increasing opportunities for empowerment (choice and control) Developing connectedness through therapeutic relationship Increasing hopefulness & optimism Strengths‐based and solution ‐focused Human‐to‐human Inspiring, mutual, non‐judgemental Learning together (different people's ideas & minds)	Intervention source (who delivers SPI) Self‐administered (digital) Clinician/psychologist/psychiatrist Peer support worker

Donabedian Model: Processes and outcomes of SPIs	
Changes related to knowledge: Being familiar with, aware of and using the intervention	
Themes	Sub‐themes
Engaging with intervention as useful resource	Reliable & consistent delivery Flexible, simple, practical intervention format Collaborating to co‐author safety plan Relevance of people and activities (i.e., personalisation)
Increasing control	Learning specific therapy models techniques and tools Increasing self‐awareness & personal insights
*Changes related to attitudes*: Including external & internal factors. Learning new facts or perspectives, direct experiences with resources of intervention, social influences, observing group norms, experiencing social pressure, understanding the underlying psychological processes that drive behaviour	
Inspiring hope	Self and others
Increasing connectedness	Identifying with intervention (for future use) ‘doing’ intervention Learning with and from others Learning explicit and tacit knowledge
Providing motivation to change	Reasons for living Therapeutic Relationship: facilitated reflection
Group norms	Peer‐to‐peer Group dynamics (inclusive & equitable/social & pleasant)
Increasing self‐awareness	Increasing self‐knowledge, insight & self‐management skills
*Changes related to behaviour*: How and why individuals modify their habits and actions to achieve goals and improve their wellbeing	
Countering suicidality	Employing strategies to counter warning sign(s) & trigger(s) Employing strategies to match or neutralise warning sign(s) & trigger(s) Employing strategies to counter emotional overwhelm Countering death wish with reasons to live Interactive distraction Maintaining wellbeing

#### 3.3.2 Stage 2

The results of the thematic analysis of the included studies themed according to CHIME recovery framework are presented in Figure 2 with number of studies in the review each theme was extracted from. (See File S4 for personal recovery results).

**FIGURE 2:**
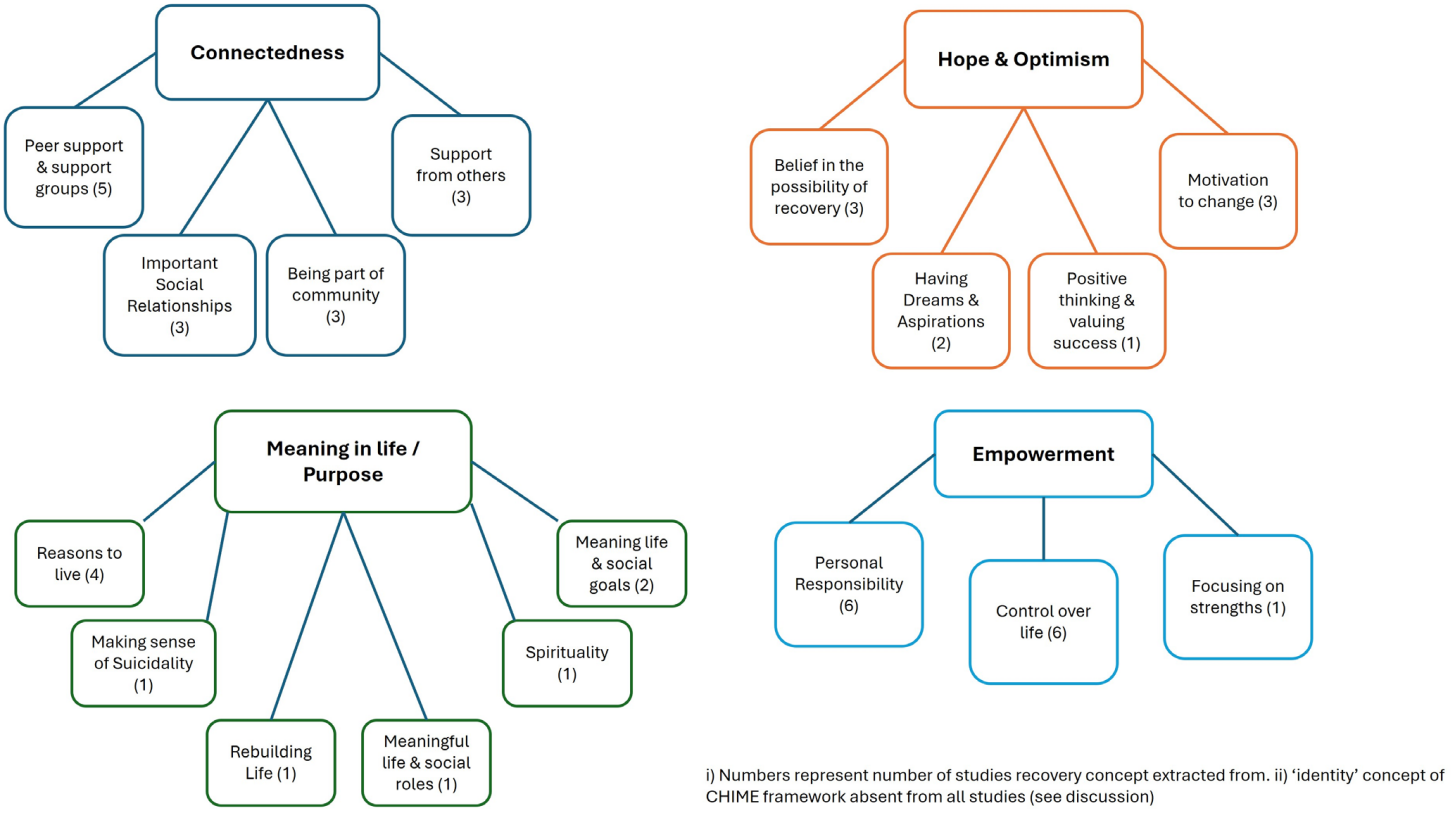
Stage 2 syntheses: Personal recovery (CHIME).

#### 3.3.3 Stage 3

The results of the translation of the individual syntheses, autoethnography, reciprocal and refutational analyses are presented in Table 6.

**TABLE 6:** Stage 3 synthesis: Translation table.

Stage 1 synthesis themes & subthemes (Donabedian & TIDieR)	Stage 2 synthesis themes & subthemes (Personal recovery)	Critical autoethnographic synthesis	Reciprocal comparison	Refutational contrast
Engaging with intervention as useful resource Reliable & consistent delivery Flexible, simple, practical intervention format Collaborating to co‐author safety plan Relevance of people & activities (personalisation)	Connectedness Peer support & support groups Important social relationships Being part of community Support from others Hope & optimism Belief in the possibility of recovery Having dreams & aspirations Positive thinking & valuing success Motivation to change Meaning in life/purpose Reasons to live Making sense of suicidality (re)building a life worth living Meaningful life & social roles Spirituality Meaning in life & social goals Empowerment Personal responsibility Control over life Focusing on strengths n.b. These themes and sub‐themes are found throughout and so do not appear here attached to any specific section.	Reached my ‘rock bottom’—I might as well try this—nothing else has worked. Clinic was difficult to get to—no public transport and I struggled to get there each time. I experienced a meaningless safety plan completed template that did not hold any particular relevance or meaning for me and I always thought was more for the clinician and organisation than for me. I put it in a drawer	Turning point in recovery narratives Written Safety plan was easy to access and refer to when needed. Acts as aide memoir	Format of delivery created some barriers to engaging and/or exacerbated suicidality. Written safety plan difficult to change as knowledge, personal insight grows. Need for flexible, adaptive template that allows for personal growth and is able to be fully personalised. Use of pre‐determined template and/or multiple‐choice options funnel people into options that aren't tailored for them.
Increasing control Learning specific therapy models, techniques & tools Increasing self‐awareness & personal insights		Learning and practicing many different therapy models DBT skills, ACT, mindfulness I discovered I wanted to use different ones on different days depending on my mood or where I was or other contexts I learned to be self‐reflective and mentalisation skills to enable me to increase my self‐awareness and personal insights	Learning specific therapy models, techniques and tools. Increasing self‐awareness & personal insights	Some formats of SPIs, that is, digital mobile phone apps, or one session SPIs lack the ability to teach but are focused on identifying what you already know. Some formats of SPIs, that is, self‐administered or single session SPIs are less enabling of increasing self‐awareness and personal insights than SPIs delivered over multiple sessions.
Inspiring hope Self and others		Until someone asked me ‘what matters to you?’ I hadn't thought about that before. Identifying my personal values helped identify my reasons to live and set some large, ambitions and dreams	Having an open mind‐set Finding reasons to live	Some SPIs do not have identifying reasons to live section. Others, downstream this as less important than other elements.
Increasing connectedness Identifying within intervention (for future use) Doing intervention Learning with & from others Learning explicit & tacit knowledge		Actively engaging with friends and family—even when feeling like I didn't want to, helped remind me of my importance in their life. This helped to counter my ‘everyone would be better off if you were dead’ thoughts New knowledge helped me be able to identify, personalise and choose self‐management strategies I was more likely to use because they fitted in with my lifestyle, personal circumstances and met my needs. As I practiced countering my suicidality, self‐growth and learning through the SPI enabled me, eventually to go beyond my suicidality and start developing my personal recovery	Meaningful social roles are seen in two ways: ‐ (1) identifying the way an individual values and connects to their own social roles (2) the other group members (in group SPIs) provided meaningful social roles for each other	
Providing motivation Reasons for living Therapeutic relationship: facilitated reflection Peer‐to‐peer Group dynamics		Learning from clinician – being able to bounce ideas of her without her problem‐solving or resorting back to unhelpful, restrictive ways of ‘keeping me safe’ Current working in peer support I understand the value of shared experiences, mutual, reciprocal, non‐judgemental support to inspire, motivate and promote active participation in SPI	Working with someone else to check out, try things, identify warning signs and what works for the individual. Providing a safe space to try out strategies to counter suicidality. When things don't work or go wrong—non‐judgemental reflection enables someone to try something else or adapt and innovate failed strategy Peer‐to‐peer sharing suicidality thoughts & experiences. Group members drew strength from each other, motivated and supported one another and learned and taught each other Some SPIs train the clinician delivering intervention to act as a facilitator of reflection and guide the user in a coaching style approach	Self‐administered SPIs (i.e., mobile phone apps) lacked this element. These safety plans generally had less personalisation The peer‐to‐peer element is missing from 1‐2‐1 and digital formats with the exception of 1‐2‐1's that are delivered by peer support workers Some studies—clinicians described workplace culture, embedded beliefs and unhelpful working practices, expectations and pressures which were barriers to delivering sensitive, gentle approach. Time pressures meant they often suggested things or gave advice and couldn't take the time to develop a good therapeutic relationship
Increasing self‐awareness Increasing self‐knowledge, insight & self‐management skills		In my experience I had to learn the difference between reflection and self‐critical. Also, when I was over analysing and it had stopped being helpful and something to learn from and had become ruminative or self‐destructive		
Countering suicidality Employing strategies to counter warning sign(s) & trigger(s) Employing strategies to match or neutralise warning sign(s) & trigger(s) Employing strategies to counter emotional overwhelm Countering death wish with reasons to live Interactive distraction Maintaining wellbeing		I didn't know what would work for me as I had never thought about that before, so I had to embark on a long process of trial‐and‐error strategies to work out what works for me, what do I like or prefer to do, how can I adapt strategies to work for me in different contexts, at different times for example I sometimes wanted time alone to wait out the suicidality and rest. Not wanting to speak or engage with anyone. But this was often a point of contention between me and my clinical team as they deemed this to be highly risky and that I was being deliberately difficult One new strategy I learned was to change my immediate environment. For example, if I'm inside and noticing suicidal thoughts I can counter them by going outside Ensuring that I have regular self‐care routines and take care of my overall wellbeing is important for maintaining my safety from suicidality	Trial‐and‐error strategies appeared in some SPIs and ways to counter suicidality described in some safety plan contents Interactive distraction found in the contents of some of the safety plans in the studies. These showed that people were engaging in activities to counter their suicidality that held their attention, rather than passively watching the TV, for example Practicing self‐care and establishing routines found in the contents of some SPIs	Not seen in SPIs that are brief, one session only, which focus on identifying and writing down ones already known or suggesting ones with multiple choice options (digital formats) Lack of getting to know the person means that some ways people can counter suicidality are more acceptable than others. This means others' fear for someone's safety may override their preference for this method of countering it. This is generally seen in SPIs that lack a focus on identifying and connecting with ‘reasons to live’

#### 3.3.4 Stage 4

Four lines‐of‐argument syntheses: (i) intervention structure and resource; (ii) changes related to behaviour; (iii) changes related to attitudes, including external & internal factors; (iv) changes related to, and ongoing pursuit of, knowledge were individually developed. These are shown together in Figure 3.

**FIGURE 3:**
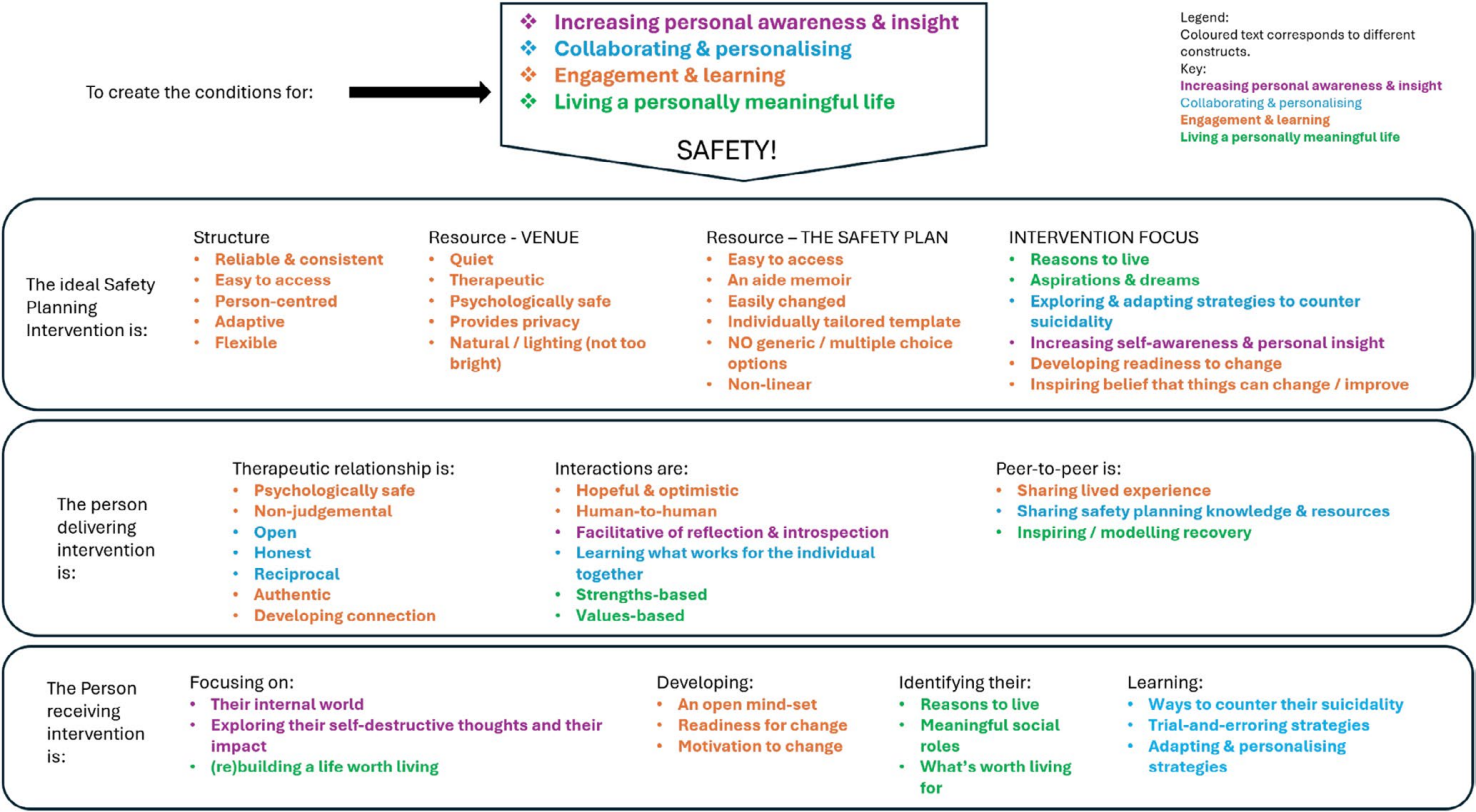
Stage 4 synthesis: Lines of argument syntheses.

#### 3.3.5 Stage 5

All the data from this review has been synthesised into three dynamic mechanisms of change—*Learning, Introspection* and *Self‐reflection—and* three interconnecting phases of personal development—*Inspiring*, *Discovering* and *Embodying—representing* broad, non‐linear periods of progression for the individual as they journey through the safety planning process. The framework foregrounds personal growth, internalisation and implementation (see Figure 4).

**FIGURE 4:**
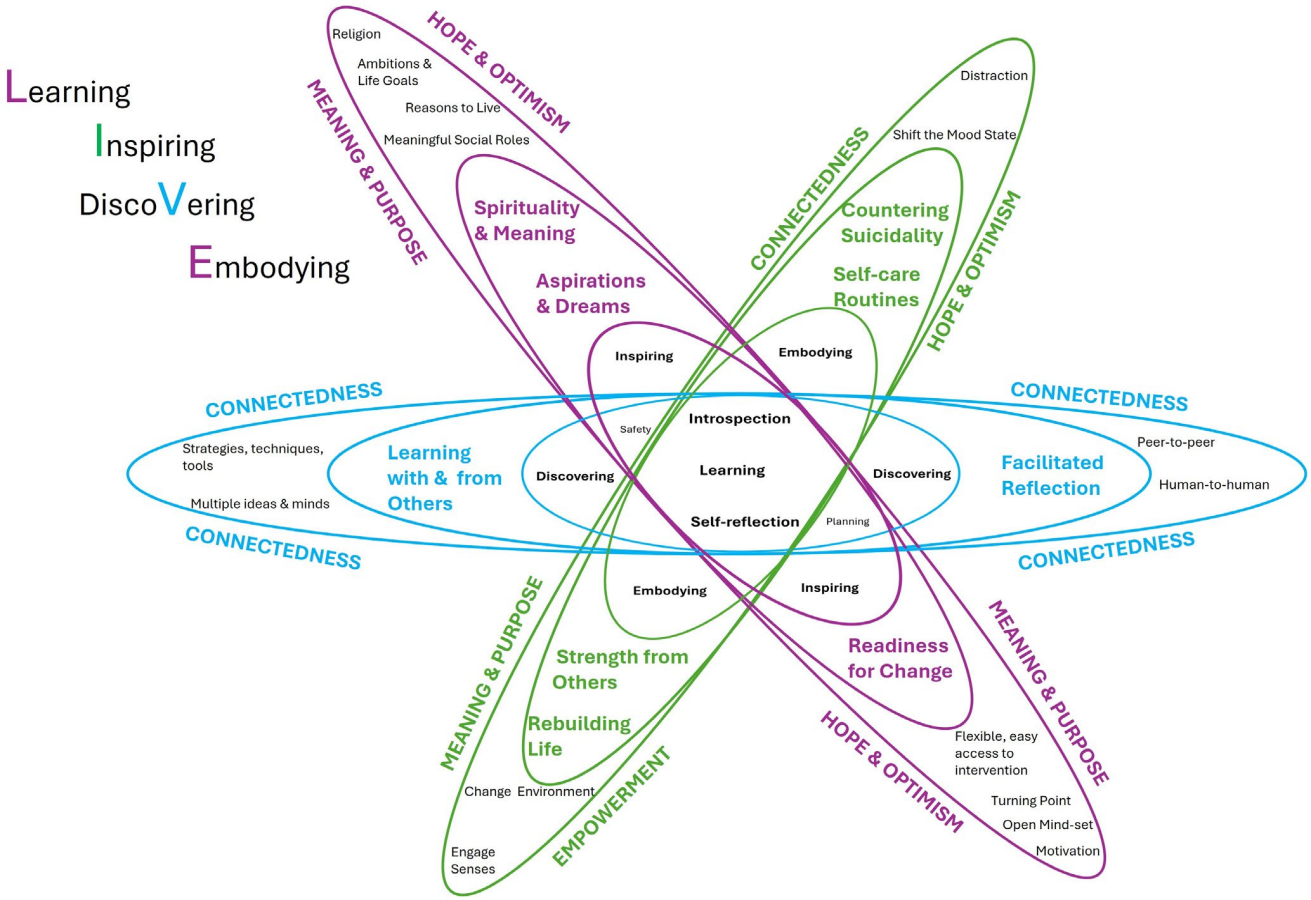
Full framework of safety planning.

##### 3.3.5.1 Mechanisms of Change

These mechanisms are cyclical, interconnected processes that operate throughout the SPI. Individuals move fluidly between *learning, introspection* and *self‐reflection* as they discover, practice and adapt behavioural change aligned with personal strengths and values. This iterative engagement fosters empowerment, self‐understanding and resilience.

1. *Learning*: The intervention provides opportunities for learning both explicit (e.g., learning specific therapeutic techniques) and implicit (e.g., sharing experiences) knowledge acquisition (Chesin et al. 2015; O'Connor et al. 2022; Porras‐Segovia et al. 2024; Rainbow, Tatnell, Blashki, and Melvin 2024; Rainbow, Tatnell, and Blashki 2024; Parrish et al. 2023).
2. *Introspection*: The intervention guides the individual to examine their internal states to understand the ‘why’ behind suicidal ideation, fostering self‐awareness and identification of warning signs (Chesin et al. 2015; Patel et al. 2023; Goodman et al. 2023; O'Connor et al. 2022; Rainbow, Tatnell, and Blashki 2024).
3. *Self‐reflection*: The intervention facilitates self‐reflection by a focus on the ‘what’ of self‐destructive behaviours and their impact on personal safety and interpersonal relationships. The intervention provides a therapeutic space for individuals to evaluate and adapt SPI resources to suit their unique experiences, circumstances, preferences and needs (Chesin et al. 2015; O'Connor et al. 2022; Patel et al. 2023).

##### 3.3.5.2 Three Phases of Personal Development

Safety planning is an intentional journey of improving skills, cultivating self‐awareness, new knowledge and better attitudes to counter suicidality in order that the individual can increase their resilience to overcome future suicidal crises. The intervention aims to enable people to realise their full potential and lead fulfilling and personally meaningful lives. Three broad, non‐linear phases of personal development were identified: (A) *inspiring*, (B) *discovering*, (C) *embodying*.

(A) *Inspiring*: marks the emotional and motivational initiation of the safety planning process. It is characterised by a renewed focus on strengths, values and meaning in life, fostering agency and hope in individuals experiencing suicidality (Shah et al. 2024). Three motivational constructs—factors influencing purposeful behaviour—were identified: (1) readiness for change; (2) aspirations & dreams; (3) spirituality & meaning in life.

1. *Readiness for change*: is a motivational construct, involving recognition of the need for transformation and a willingness to act. Emotional‐cognitive activation may be necessary to overcome impaired problem‐solving during suicidal crises (Rainbow, Tatnell, and Blashki 2024). These were synthesised into the following sub‐themes:
  - *Turning point*: a shift in narrative or mindset signalling readiness to engage (for e.g., O'Connor et al. 2022).
  - *Open mind‐set*: receptiveness to new ideas and perspectives, often catalysed by suicide‐related crisis (Chesin et al. 2015; O'Connor et al. 2022).
  - *Motivation to change*: driven by both extrinsic (e.g., professional support or intervention resources) and intrinsic (e.g., rooted in hope) factors (Chesin et al. 2015; Shah et al. 2024; Melvin et al. 2019).
  - *Flexible, easy access to SPI*: accessibility of SPIs is critical, with barriers such as mental health symptoms and life demands impacting engagement (Patel et al. 2023; Melvin et al. 2019; O'Connor et al. 2022).
2. *Aspirations and dreams*: conceptually linked to personal recovery concepts *hope and optimism*, contribute to wellbeing and treatment motivation (Wong and Fry 1998; Nair 2003). The synthesis revealed two interrelated sub‐themes:
  - *Ambitions and life goals*: future orientated thinking persists even during suicide‐related crisis reflecting underlying motivation and hopefulness (Porras‐Segovia et al. 2024).
  - *Reasons to live*: articulating personal motivation(s) for living enhances engagement and emotional investment in the SPI (Rainbow, Tatnell, and Blashki 2024; Patel et al. 2023; Porras‐Segovia et al. 2024; Melvin et al. 2019)
3. *Spirituality and meaning in life*: defined here as ‘the search for meaning in life and connection beyond distress’. Making this explicit through the SPI supports personal recovery, meaningful engagement in the SPI and motivation to make behavioural change. The synthesis revealed two sub‐themes:
  - *Religion*: faith can affirm self‐worth and resilience, offering strength, inspiration and motivation to engage with the SPI (Rainbow, Tatnell, and Blashki 2024).
  - *Meaningful social roles*: valued roles such as parenting or caregiving motivate engagement and use of safety planning to maintain safety (Rainbow, Tatnell, and Blashki 2024)

(B) *Discovering*: Characterised by personal learning and growth. Individuals explore their unique experiences and circumstances and how these shape decision‐making and behaviour. This understanding empowers individuals to be able to make informed decisions about safety behaviours, tailoring intervention resources to meet their individual preferences and needs. Two collaborative processes underpin this phase: (1) learning with and from others; (2) facilitated reflection.

1. *Learning with and from others*: Peer support and therapeutic alliance facilitate mutual learning and validation. The synthesis revealed two sub‐themes:
  - *Different people's ideas and minds*: exposure to diverse perspectives by others participating in and those delivering the SPI fosters new thinking (Goodman et al. 2020; Rainbow, Tatnell, and Blashki 2024; Rainbow, Tatnell, Blashki, and Melvin 2024; O'Connor et al. 2022; Wilson et al. 2022; Melvin et al. 2019; Shah et al. 2024).
  - *Peer‐to‐peer*: shared lived experience builds trust and enhances receptivity to change (Goodman et al. 2023; Patel et al. 2023; Vijayakumar et al. 2017; Wilson et al. 2022).
2. Facilitated reflection: a reimagining of the clinician's role as ‘facilitator’ guiding service users through an intentional process of introspection and self‐reflection. Through the creation of a safe, validating therapeutic relationship, individuals are encouraged to explore their suicidality, expressing what matters most to them. This reflective dialogue fosters deeper understanding and self‐awareness. The synthesis revealed two sub‐themes:
  - *Human‐to‐human*: emphasising relational depth and mutual respect in therapeutic interactions (O'Connor et al. 2022; Shah et al. 2024).
  - *Acquisition of knowledge and skills*: individuals develop practical techniques and strategies through the intervention resources to manage their suicidality (Chesin et al. 2015; Melvin et al. 2019; Patel et al. 2023; Porras‐Segovia et al. 2024; Shah et al. 2024).

(C) *Embodying*: reflects the integration of knowledge and concepts into ‘living’ experience as individuals begin to internalise the SPI resources and learning, transforming it into knowledge that informs everyday decision‐making and behaviour. This phase supports the transition from ‘planning’ into ‘action’, as individuals focus on (re)building personally meaningful and fulfilling lives. Four safety planning strategies underpin this phase: (1) countering suicidality; (2) self‐care routines; (3) drawing strength from others; (4) (re)building a life worth living.

*Countering suicidality*: the ability to oppose or nullify the effect of suicidality by doing an opposing action, resisting acting in self‐destructive and harmful ways, and implementing new safety behaviours learned in the SPI. The synthesis revealed there were five ways people were countering suicidality reported in the safety plans:
  - *Shift the mood state*: refers to the intentional use of technology, for example, ‘watch stand‐up comedy shows’ (Rainbow, Tatnell, and Blashki 2024) or using a specific therapy technique taught in an intervention for example mindfulness (Chesin et al. 2015) to shift the mood when in the midst of experiencing suicidal thoughts and feelings.
  - *Interactive distraction*: being actively engaged in a distracting activity as a way to self‐manage intense emotions, painful experiences and a way to counter the rumination and intensity of suicidal ideation (Porras‐Segovia et al. 2024; Rainbow, Tatnell, Blashki, and Melvin 2024).
  - *Engaging the senses*: all of the five senses are used for example smell (lighting a scented candle); taste (bake something sweet); touch (snuggle under a weighted blanket) (Rainbow, Tatnell, and Blashki 2024).
  - *Changing the environment*: refers to modifying an existing space (‘make my room feel calmer—light a candle’) or deliberately seeking out a change of en vironment (Rainbow, Tatnell, and Blashki 2024).
  - *Mind–body relationship*: countering emotional overwhelm by physical activity (e.g., stretching, dancing, lifting weights (Rainbow, Tatnell, and Blashki 2024)).
*Self‐care routines*: establishing and maintaining self‐care routines that support emotional and physical wellbeing (Chesin et al. 2015; O'Connor et al. 2022; Rainbow, Tatnell, and Blashki 2024; Shah et al. 2024).
*Drawing strength from others*: Sustaining recovery through relational support and connectedness (Rainbow, Tatnell, Blashki, and Melvin 2024; Goodman et al. 2023; Chesin et al. 2015).
*(Re)building a life worth living*: personal recovery is foregrounded as individuals pursue goals and values that foster meaning and hope and improve quality of life (Rainbow, Tatnell, and Blashki 2024; Patel et al. 2023; Porras‐Segovia et al. 2024; Melvin et al. 2019).

## 4 Discussion

This integrative literature review, enriched by lived experience, presents a novel, empirically based framework for safety planning that foregrounds personal recovery. The synthesis of evidence directly addresses the review's two questions and offers critical insights into the conceptual underpinnings of safety planning interventions.

*What SPIs have been developed and how do they work for adults experiencing suicidal ideation and/or self‐harming behaviours to increase physical and psychological safety?*

The review identified significant heterogeneity in SPI design, delivery formats and target populations which limits generalisability of findings. All interventions in this study build on the original safety planning intervention (Stanley and Brown 2012), which promotes a sequential, stepped safety plan approach, however, this model assumes a linear progression of suicidality organised along a hierarchical continuum—a view challenged by both AG and by substantial empirical evidence (for e.g., see Ursano et al. 2015; Nock et al. 2008). The new recovery‐orientated framework proposed here addresses this by reconceptualising safety planning as dynamic, non‐linear processes of personal development. It introduces three interconnected phases—*inspiring*, *discovering* and *embodying—*which reflect motivational, reflective and behavioural dimensions of safety planning. These phases challenge the dominant biomedical and risk‐management paradigms by emphasising relational, personalised and recovery‐focused approaches. The framework also repositions the clinicians' role from ‘*risk manager*’ to ‘*facilitator*’, supporting individuals to engage meaningfully with safety planning resources and processes. This reconceptualization recognises that, in relation to suicide prevention, ‘relational care’ is poorly defined and requires further co‐produced research to identify its core components and develop a clear framework for its application and evaluation’ (Griffiths et al. 2025). The framework identifies three mechanisms of change—*learning, introspection* and *self‐reflection*—across all its phases. These mechanisms operate cyclically and support individuals to develop clarity, adapt strategies and align behaviours with personal values.

The majority of included studies excluded individuals with severe mental illness (SMI) participating in safety planning, despite policy recommendations advocating SPI use in psychiatric services. This exclusion significantly undermines the evidence for SPI use with this population. The autoethnographic synthesis enriched this understanding by highlighting how lived experience can inform and deepen our knowledge of SPIs, particularly for individuals navigating complex emotional and psychological needs.

*What is the relationship between conceptual and/or theoretical understandings of how SPIs work and how personal recovery concepts and theoretical understandings work to increase safety behaviours and outcomes?*

The review applied the CHIME framework (Leamy et al. 2011) to analyse personal recovery processes within SPIs. While most studies demonstrated elements of *connectedness*, *hope*, *meaning* and *empowerment*, the concept of *identity* was notably absent. This omission underscores the lack of focus on personal recovery of the SPIs in this review, which prioritise clinical recovery (the decrease or absence of symptoms), rooting them firmly in biomedical understandings of mental health. The new framework's emphasis on motivational constructs such as *aspirations and dreams*, and *spirituality and meaning in life*, addresses a critical gap in the SPI literature. These constructs are central to personal recovery but are often absent in traditional safety planning interventions. By integrating these elements, the framework offers a more holistic approach to suicide prevention.

This review highlights a critical knowledge gap in the current SPI literature—the limited integration of personal recovery principles and lived experience perspectives in the conceptualisation, design and evaluation of SPIs. Despite policy endorsements for recovery‐orientated approaches in suicide prevention (e.g., World Health Organization 2024), most studies reviewed lacked co‐production, excluded individuals with SMI and failed to incorporate concepts central to personal recovery. The absence of the *identity* domain from the CHIME framework in the reviewed studies further underscores this gap. Moreover, the SPI literature tends to prioritise clinical control or friends and family support over empowering personal autonomy through a process of learning and personal growth. This review challenges the assumption that suicidality is a predictable, sequential process by reconceptualising safety planning as a dynamic, non‐linear process of personal development requiring relational depth and adaptation of intervention resources to suit the unique experiences, circumstances, preferences and needs of every individual who encounters it.

By proposing a new empirically based, recovery‐orientated framework, this review addresses the knowledge gap by:

- Reframing SPI delivery around personal development phases (*inspiring, discovering, embodying*)
- Identifying mechanisms of change (*learning, introspection, self‐reflection*)
- Highlighting the importance of therapeutic relationships and expert facilitation
- Emphasing the need for co‐production and lived experience integration in future SPI research and practice.

This framework offers a more nuanced and responsive approach to suicide prevention, particularly for individuals with enduring suicidality and complex mental health needs and lays the groundwork for future evaluations and co‐produced interventions. A key contribution of this review lies in its integration of diverse concepts, theories and empirical findings to construct new, recovery‐orientated understandings of SPIs. The synthesis process drew on multiple theoretical frameworks—including the Donabedian Model of healthcare quality, the CHIME framework of personal recovery and meta‐autoethnographic synthesis methods—to interpret and reframe the literature through a lived experience lens.

The Donabedian Model enabled a structured analysis of SPI components, focusing on structure, processes and outcomes within healthcare delivery. The CHIME framework complemented this by offering a conceptual scaffold for understanding personal recovery processes. The researchers applied both frameworks to verbatim data extracted from the studies, enabling a multifaceted synthesis that revealed where recovery principles were present, and where they were absent, in existing SPI designs. Through synthesis, the review identified that while many SPIs are structurally sound (e.g., clear components, delivery formats), they often lack depth in processes, particularly relational and reflective elements, and fail to capture meaningful outcomes beyond reduction in suicide‐related symptoms. The synthesis demonstrates that quality in suicide prevention is not solely defined by clinical outcomes (e.g., reduction of symptoms or pathology), but also by the presence of non‐judgemental, reciprocal therapeutic relationships, personalisation and empowerment. The new framework addresses this by:‐

- Reframing SPI processes as relational and service‐user led, rather than transactional and clinician led.
- Expanding outcome measures to include personal growth, agency and therapeutic engagement, which are more aligned with recovery‐orientated care.
- Highlighting the importance of flexibility and adaptability in intervention structure and resources to meet each individual's' unique experiences, circumstances, preferences and needs.

The CHIME framework was used to explore how SPIs support recovery processes. The synthesis revealed that while *connectedness, hope, meaning* and *empowerment* were present in some studies, *identity* was notably absent. The review extends CHIME's applicability to suicide prevention, showing how recovery principles can be operationalised within SPIs to support personal recovery understandings of individuals' experiences of suicidality. The new framework contributes to CHIME by:

- Introducing motivational constructs (e.g., inspiring) that deepen the understanding of hope and meaning.
- Emphasising identity formation through self‐reflection and introspection
- Demonstrating how peer support and expert facilitation foster connectedness and empowerment in ways not captured by traditional SPIs.

The integration of these frameworks was further enriched by the autoethnographic synthesis, which introduced a critical, experiential dimension to the analysis. This approach enabled the identification of motivational constructs that are overlooked in SPI literature but are central to personal recovery. The lived experience perspective also challenges dominant assumptions in the literature, such as the hierarchical continuum of suicide risk. Through reciprocal and refutational syntheses, the review juxtaposed empirical findings with autoethnographic insights to identify points of convergence and divergence. This process culminated in the development of a new theoretical framework comprising three phases of personal development—*inspiring, discovering* and *embodying*—and three dynamic mechanisms of change—*learning, introspection* and *self‐reflection*. These elements were not derived from any single study but emerged from the integration of multiple perspectives, methodologies and theoretical orientations.

The use of reciprocal and refutational synthesis adapted from meta‐ethnography enabled the integration of diverse study findings with lived experience understanding. This methodological innovation:

- Positioned lived experience as a legitimate and critical source of theoretical development
- Validated the diversity of experiences in SPI engagement
- Allowed for the construction of a higher‐order interpretation—the three‐phase framework (*inspiring, discovering, embodying*) that transcends individual study limitations.

The adaptation of meta‐ethnographic synthesis to include autoethnographic insights represents a methodological and theoretical innovation. By integrating lived experience into the synthesis process, the review challenges gtraditional hierarchies of evidence and proposes a more inclusive approach to theory‐building. This positions autoethnography not only as a reflective tool but as a legitimate contributor to theoretical development in mental health research.

The review culminates in the development of a new, empirically based, recovery‐orientated framework for SPIs. This framework introduces three phases of personal development—*inspiring, discovering and embodying*—and three dynamic mechanisms of change—*learning, introspection and self‐reflection*. These constructs are not present in existing SPI models and represent a novel theoretical contribution that integrates clinical, experiential and recovery‐orientated perspectives. The framework offers a new lens through which to understand how individuals engage with safety planning and how interventions can be co‐produced to decrease suicide‐related risks and increase personal growth, recovery and overall wellbeing.

### 4.1 Strengths and Limitations

#### 4.1.1 Strengths

*Novel methodological integration*: This review is the first to combine an integrative literature review with a critical meta‐autoethnographic synthesis, offering a unique methodological contribution that foregrounds lived experience in the analysis and interpretation of SPIs.

*Development of a new framework*: The synthesis led to the creation of a new, empirically based, recovery‐orientated framework for SPIs, which integrates personal recovery principles and offers practical guidance for clinical application.

*Use of multiple theoretical models*: The review draws on established frameworks—Donabedian, CHIME, TIDieR, MMAT, PRISMA—and applies them innovatively to suicide prevention, enhancing their relevance and utility in suicide research.

*Rich lived experience perspective*: The autoethnographic component adds depth and authenticity to the synthesis, challenging dominant biomedical assumptions and offering insights that are often excluded from traditional reviews.

*Structured & transparent synthesis*: The five‐stage synthesis process is clearly articulated and replicable, enhancing transparency and methodological rigour.

#### 4.1.2 Limitations

*Single reviewer for key stages*: The literature search, study selection and data extraction were primarily conducted by one reviewer, which may introduce bias. The research team discussed and reviewed all stages collaboratively to mitigate this risk; however, findings should be interpreted with caution.

*Language and publication bias*: The review was limited to English‐language peer‐reviewed studies, potentially excluding relevant research published in other languages or grey literature.

*Exclusion of certain populations*: The review excluded studies focused on children, individuals with neurodiversity, learning disabilities and forensic populations. Future research should explore the framework's applicability for these groups.

*Conceptual limitations of autoethnography*: Whilst autoethnography enriches the synthesis, it prioritises idiographic knowledge, which may not be generalisable. This tension between individual insight and broader applicability is acknowledged and embraced as part of the review's epistemological stance.

*Limited empirical testing*: The proposed framework has not yet been empirically tested. Its effectiveness and applicability in clinical settings remain to be evaluated through future research.

### 4.2 Implications for Practice

The findings of this review have significant implications for clinical practice, particularly within mental health nursing, psychiatry and lived experience workforces. The proposed recovery‐orientated safety planning framework offers a practical, evidence‐based model that can enhance the therapeutic value of SPIs and improve outcomes for people experiencing suicidality.

*Reframing the clinician's role*: The review reconceptualises the clinicians' role from ‘risk manager’ to ‘facilitator’. This shift encourages practitioners to move beyond checklist‐based crisis plans and instead engage in relational, reflective conversations that support meaning making, agency, learning and recovery. Training programmes should incorporate this reframing to help clinicians develop skills in developing psychologically safe therapeutic relationships, and expert facilitation techniques because relational depth is central to the effectiveness of SPIs. The framework underscores the value of non‐judgemental, validating therapeutic relationships in fostering engagement and behavioural change. Mental health services should invest in staff development that promotes empathy, active listening personalisation of treatment, collaboration and personal recovery.

*Personalisation of safety planning*: The framework emphasises the importance of tailoring SPIs to the individuals' unique experiences, circumstances, preferences and needs. This includes integrating ‘personal values’, ‘aspirations’ and ‘reasons to live’ into safety plans. Practitioners should co‐create safety plans with service users, using flexible formats and culturally sensitive approaches that reflect the diversity of suicidal experiences.

*Supporting individuals with SMI*: The review highlights the exclusion of people with SMI from much of the SPI literature and practice. Services should prioritise inclusive practices and ensure that SPI delivery is easy to access, flexible in format and delivery, and fully co‐produced.

*Utilisation of peer support*: This framework can be used to develop training for this workforce in SPI delivery. Services should invest in peer support workers, particularly in inpatient and crisis settings, to deliver recovery‐orientated SPIs.

*Moving beyond risk reduction*: Traditional SPIs focus narrowly on reducing suicidal ideation or behaviour. The new framework expands the scope to include personal development, emotional regulation and life rebuilding. Practitioners should be encouraged to view safety planning as a process of growth rather than a static intervention, and to measure outcomes that demonstrate relational care and personal recovery effectiveness.

*Alignment with policy and guidelines*: The framework aligns globally with WHO suicide prevention guidance (World Health Organization 2024) and NHS England's ‘staying safe from suicide’ guidance (NHS England 2025) and provides a practical tool for implementing these recommendations in clinical settings. It can inform service redesign, workforce training and quality improvement initiatives.

## 5 Conclusion

This integrative literature review and critical meta‐autoethnographic synthesis offer a new recovery‐orientated framework for safety planning interventions that challenge dominant biomedical models and reframe safety planning as a dynamic, relational and personalised process of change towards improving quality of life. By synthesising the evidence with lived experience insights, the review highlights a critical gap in much SPI design—particularly the lack of co‐production, exclusion of delivery to individuals with SMI and limited integration of personal recovery principles. The proposed framework introduces three phases of personal development and three mechanisms of change, offering a theoretically grounded and practice‐informed model for delivering current global and UK policies and guidance for suicide prevention. This work contributes to the advancement of recovery‐orientated suicide prevention and provides a foundation for future research, clinical innovation and policy development.

## 6 Relevance Statement

### 6.1 What is Known on the Subject

- Safety planning interventions are widely used in suicide prevention but often lack personalisation and recovery‐orientated approaches.
- Existing safety planning interventions are typically brief, clinician‐led and based on biomedical assumptions.

### 6.2 Originality

- This review is the first to integrate lived experience of suicidality and safety planning use into a comprehensive analysis of SPIs.
- It proposes a new, empirically based, recovery‐orientated safety planning framework that emphasises personal development, therapeutic relationships and individual agency.

### 6.3 Significance

- The findings offer practical guidance for mental health professionals to deliver more personalised and relational safety planning care.
- The framework supports inclusive, recovery‐focused care for individuals with SMI and enduring self‐destructive behaviours.
- It provides a foundation for policy implementation, service redesign, quality improvement and workforce training aligned with global and UK suicide prevention guidance.

## Author Contributions

**Amanda Kate Green:** conceptualisation (lead); formal analysis (lead); investigation (lead); methodology (equal); project management (lead); validation (equal); visualisation (lead); writing original draft (lead); writing – review and editing (equal). **Alan Simpson:** conceptualisation (supporting); formal analysis (supporting); methodology (equal); supervision (supporting); validation (equal); visualisation (supporting); writing – review and editing (equal). **Vasiliki Tzouvara:** conceptualisation (supporting); formal analysis (supporting); investigation (supporting); supervision (supporting); validation (equal); visualisation (supporting); writing – review and editing (equal). **Corinna Hackmann:** methodology (equal); supervision (supporting); visualisation (supporting); writing – review and editing (equal). **Eva Klotz:** investigation (supporting); **Mary Leamy:** conceptualisation (supporting); formal analysis (supporting); supervision (lead); validation (equal); visualisation (supporting); writing – review and editing (equal).

## Funding

The authors have nothing to report.

## Ethics Statement

This paper is part of a larger realist evaluation of safety planning which aims to develop realist programme theories of safety planning to guide the development of SPIs for people with SMI. The study has ethical approval IRAS293012, is being conducted in an NHS mental health trust, and is sponsored by King's College London.

## Reflexivity Statement

Critical meta‐autoethnographic synthesis required X to reflect upon and use her personal experience of using a SPI to counter her lived experience of enduring suicidality and SMI. The autoethnography was not analysed in isolation; rather, X used her lived experience to relate to and interpret the individual studies, comparing and contrasting concepts, themes and interpretations to identify how they related to (or not) her experiences. To be as robust, transparent and rigorous as possible in the application of her lived experience to this research, a reciprocal and refutational synthesis was produced, adapted from meta‐ethnography methodology (Noblit and Hare 1988). The translation table has a separate column for X's lived experience (see Table 5). Ethical considerations when using her lived experience of suicidality were considered to ensure she was not causing harm to herself through her reflections. A balance was struck between personal reflection and rigorous analysis, ensuring that the autoethnographic elements shared are only ones which are relevant and can contribute to a well‐developed and insightful literature review. X constantly examined her own role in the research process and discussed this with her co‐authors. This has led to a more critical and nuanced analysis of the literature on SPIs. Throughout she remained constantly aware of her own biases and how these might be influencing her interpretations. She reflects on her own experiences of suicidality and the personal growth process required to change her responses to it, as well as those of others she has supported working in peer support, in order to develop her safety planning knowledge. These reflections shape her understanding throughout this review. Where her personal experiences differ from other users of safety planning reported in these studies, this is embraced and all perspectives are integrated into the framework. This provides the underlying philosophy of the interpretation—that no individuals' experiences are universal experiences. This is also a fundamental tenet of the underlying philosophy of the safety planning framework.

## Conflicts of Interest

The authors declare no conflicts of interest.

## Supporting information


**File S1:** Critical Meta‐Autoethnographic Synthesis (CMAS) Methodology.

**File S2:** Completed TIDieR Checklist included studies.

**File S3:** Stage 1 synthesis Donabedian Model.

**File S4:** Personal recovery results.

## Data Availability

The data that supported the findings of this study are available from the corresponding author upon reasonable request.
