# Supplementary material for: An Empirically Based, Recovery‐Orientated Framework for Safety Planning Interventions for Suicide Prevention: An Integrative Literature Review and Critical Meta‐Autoethnographic Synthesis
Source: J Psychiatr Ment Health Nurs. 2026 Sep 9;33(6):1060–81. doi: 10.1111/jpm.70202 (PMC13748692; doi:10.1111/jpm.70202)
Supplement: Supplementary file 1 [file JPM-33-1060-s001.docx]

**Supplementary File S1. Critical Meta-Autoethnographic Synthesis (CMAS) Methodology**

Critical meta-autoethnographic synthesis (CMAS) is an interpretive, inductive qualitative synthesis methodology designed to rigorously and transparently integrate the lived experience of the researcher into the analytic process. While lived experience research has a long history with significant progress being made to establish the value of lived experience research in mental health and clinical research fields (for e.g. see (Deegan, 2007; Morrell-Bellai & Boydell, 1994), there remains no clear strategy for guiding analysis, synthesis and conclusion-drawing when the researcher leading the study is the holder of that lived experience. CMAS adapts and extends Noblit & Hare’s (1988) meta-ethnography to address this gap, offering a structured, seven-stage approach that intentionally incorporates lived experience both as data and as an interpretive lens, without compromising quality, transparency or replicability. In doing so, it provides a practical ‘how-to’ framework for lived experience-led research that balances creativity with scientific rigour.

**How critical meta-autoethnographic synthesis adapts meta-ethnography**

| **Meta-ethnography (Noblit & Hare, 1988)** | **Critical meta-autoethnographic Synthesis (CMAS)** |
| --- | --- |
| **Definition:** An interpretive and inductive synthesis methodology | **Definition:** An interpretive and inductive synthesis methodology that rigorously and transparently integrates lived experience-led research |
| **Core processes:** Translation of studies into one another | **Core processes:** Translation of researchers’ lived experience (LEX) and included studies into one another |
| **Purpose:** a seven-phases theory-based and potentially theory-generating approach to qualitative evidence-synthesis | **Purpose:** A seven-phase, theory-generating approach for synthesising lived experience-led research evidence with other qualitative evidence. CMAS is specifically for situations where the researcher conducting the study has lived experience relevant to the topic |
| **Eligibility:** designed for synthesising qualitative studies | **Eligibility:** designed for integrating lived experience-led research, not for participatory research, lived experience advisory groups or co-design, where lived experience is relevant to the topic being studied. |
| **Phases:**   1. Selecting meta-ethnography 2. Deciding what is relevant 3. Reading and appraising studies 4. Determining how studies are related 5. Translating studies into one another using reciprocal and refutational translations 6. Synthesising translations 7. Expressing the synthesis | **Phases:**   1. Selecting critical meta-autoethnographic synthesis and primary methodology for study 2. Conducting the study using the primary methodology 3. Deciding what part of the researchers’ lived experience is relevant: distinguishing between LEX as data; LEX as interpretive lens 4. Determining how studies and LEX are related (LEX data & LEX as interpretive lens) 5. Translating LEX data and studies into one another using reciprocal and refutational translation 6. Synthesising translations: using lines-of-argument (LEX as interpretive lens) 7. Expressing the synthesis with LEX as interpretive lens |

Critical meta-autoethnographic synthesis builds directly on the logic of meta-ethnography but introduces a major methodological innovation – the intentional, structured integration of the researcher’s own lived experience. Where meta-ethnography translates studies into one another CMAS translates the lived experience of the researcher and the studies into one another, treating lived experience as both a source of data and as an interpretive lens. This requires additional steps – such as defining criteria for what aspects of lived experience are relevant and establishing transparent procedures for reciprocal and refutational translation between lived experience and study findings. CMAS retains the theory-generating power of meta-ethnography while addressing a long-standing gap in lived experience research – the absence of a practical, rigorous guide for researchers who bring their own lived experience to the analytic process.

**Strengths and Limitations**

As an innovative, novel methodology, critical meta-autoethnographic synthesis requires further testing across diverse research contexts to refine its procedures and evaluate its robustness. Its key strength lies in offering a transparent, theory-generating structure for integrating relevant lived experience in lived experience-led research without compromising rigour, addressing a gap where the complexity inherent in combining diverse methodologies runs the risk of inaccuracy and bias. At the same time, CMAS is still in its developmental phase, and ongoing methodological scrutiny is needed to ensure consistency, replicability and clarity around how lived experience is selected, expressed distinctly as either data or interpretive lens, positioned and translated within the synthesis.

**Future Research plans**

Following inclusion into my PhD thesis which will include a methodology section that will articulate critical meta-autoethnographic synthesis in greater depth.

A full methodology paper will be written that articulates critical meta-autoethnographic synthesis in greater depth, including worked examples and guidance for researchers applying it in different contexts.

Empirical testing across multiple studies will be essential to evaluate its consistency, refine its procedures and strengthen the evidence base for integrating lived experience in lived experience-led research as both data and interpretive lens. This next phase of methodological development will help establish CMAS as a robust, transferable approach within lived experience-led research.
