# Supplementary material for: An Empirically Based, Recovery‐Orientated Framework for Safety Planning Interventions for Suicide Prevention: An Integrative Literature Review and Critical Meta‐Autoethnographic Synthesis
Source: J Psychiatr Ment Health Nurs. 2026 Sep 9;33(6):1060–81. doi: 10.1111/jpm.70202 (PMC13748692; doi:10.1111/jpm.70202)
Supplement: Supplementary file 2 [file JPM-33-1060-s002.docx]

| **Supplementary File S3. Stage 1 synthesis Donabedian Model** | | | |
| --- | --- | --- | --- |
| **Donabedian Model Category** | **Themes** | **Sub-themes** | **Example quotes in the literature** |
| Changes related to knowledge: being familiar with, aware of and using the intervention | Engaging with intervention as useful resource | Reliable & consistent delivery | “I get undivided attention. Because I know that there's a guarantee that I'll have a session that Wednesday” (Patel, p.277).  Participants commented on the reliability and consistency that PLF-T offered. (Patel, p.275). |
|  |  | Flexible, simple, practical intervention format | ‘Most participants used the app to make changes to their safety plan (77.3%)’ (Melvin p.852)  Feature of the intervention style that they found to be important facilitators of initial and ongoing engagement. Most frequently, these centred around the level of flexibility, simplicity and practicality that the intervention offered and where this suited individuals' needs particularly well. "everything was just there for you, on one sheet of paper, or on your phone, you could have it with you all the time, if you felt you needed it... I've never felt it was difficult to use. I thought the way it was laid out was really good, had all the information there. The stress is kind of off you, as soon as you got that, a lot of it" (O’Connor p.9)  Utility captured the app accessibility e.g. "it's easy to access and always with me"; "having a plan on you at all times"), and ease of use "I love how everything is so easy to access", "simplicity", "the straightforwardness of the layout" (Melvin p.852) |
|  |  | Collaborating to co-author safety plan | Half of the participants self-authored their plans (n=67, 50.8%). Most who co-authored their plans worked with health professionals or support workers (n=60, 45.5%) (Rainbow p.297)  ‘The quality and completeness of the safety plans significantly varied between the two groups, with patients in the peer-delivered condition having more complete and higher-quality plans. The time to make the safety plan increased in the peer group, but this increase did not significantly affect time to disposition or total ED LOS’ (Wilson p.1090) |
|  |  | Relevance of people and activities (i.e. personalisation) | ‘During the (follow-up) telephone calls 81% of participants discussed the content of their safety plan with the intervention researchers and reflected on the relevance of any people and activities they had listed on the SP’(O’Connor p.8).  Overall plans demonstrated a high level of personalisation with respect to original rather than app-generated content. All participants were able to name at least one warning sign of an impending suicide crisis (step 1). Step 3 (reasons for living) was the most personalised section, with over two thirds of participants using wholly original text. Half of all participants (n=75, 50.0%) chose to upload photos or videos into their plan, chiefly in the reasons for living section. ( Rainbow (1) p.367)  customizability e.g. customising the safety plan" "being able to change things when I needed" (Melvin, p.852) |
|  | Increasing control | Learning specific therapy models techniques and tools | Participant quote: "The mindfulness helps me to stay above the negativity better than traditional therapy or medication (Chesin p.1351) |
|  |  | Increasing self- awareness & personal insights | Participant quote: "It has helped me to be more mindful of my emotions and not to let them consume me (Chesin, p.1351)  Participants entered between 2.78 and 6.28 entries, on average, for the warning signs, reasons for living, making the environment safe, and internal coping strategy sections (Melvin p.852)  Increased awareness of suicidal thoughts and behaviours. A majority of participants commented on the group and its impact on building awareness of their thoughts and feelings in a time of crisis. Participants reported that the groups helped them learn to identify warning signs and understanding the connection between their depression, PTSD and substance use disorder and suicidal thoughts, urges, and plans. (Patel, p.277).  Some patients reported that they used the safety plan once and subsequently applied its content from memory without activating it (Porras-Segovia, p.287)  "So it helped prepare me a little bit, helped prepare me a little bit more" (Patel P278). |
| Changes related to attitudes: including external and internal factors. Learning new facts or perspectives, direct experiences with resources of intervention, social influences, observing group norms, experiencing social pressure, understanding the underlying psychological processes that drive behaviour | Inspiring Hope | Self and others | Hope demonstrated by quotes such as "including reasons to live", "It's good to look at when you feel as though there's no hope" and "the things to live for" (Melvin, p.852)  Patients described how the intervention gave them both hope that things would improve and strategies to put in place. This gave them the agency to improve their mental health, manage and prevent future crises 'she gave me, you know, not false hope, that everything suddenly will change. But she gave me like a tool that I can you know, I can... I'm resilient enough to go and you know, continue to fight for my health. and so yeah she gave me this tool that it's really, really, really positive (Shah p.5) |
|  | Increasing connectedness | Identifying within intervention (for future use) | Connection was illustrated by "Something to talk about with friends", "being able to send your safety plan to people" and "When you can't think clearly, you can look at the app and see who's there to get help from" (Melvin, p.852) |
|  |  | ‘Doing’ intervention | Beyond using the safety plan itself, participants also reported finding a benefit from being able to 'off-load' or 'talk about how they were feeling in the days and weeks after their suicide attempt and from the sense of knowing someone was available to support them at this time' (O’Connor p.9)  Enabling catharsis and reducing loneliness through having someone to listen and talk to (Shah, p.7) |
|  |  | Learning with and from others | Veteran history of suicide attempt was associated with greater declines in caregiver burden over the study. This indicates supporting partners of Veterans with a history of attempt showed general adaptation to the veterans' elevated risk (Goodman 2021, p.53).  A majority of participants reported that they were able to open up and disclose their suicidal thoughts to the group or other support persons, share details about emotionally challenging periods and ask for group input on problems that wanted help in solving (Patel p.277)  Practitioners and patients described a joint exploration of the patients' journey and joint creation of plans together. The give and take between practitioner and patient opened up a two-way learning process which was respectful of the patient's knowledge and experience (Shah p7)  Positive interactions increased participants' motivation to engage with the processes of safety planning. (O’Connor p.10). |
|  |  | Learning explicit and tacit knowledge | "mindfulness is a useful technique to learn, even if it's not a panacea' (Chesin p.1351).  Favourite functions that participants liked the most was internal coping strategies and personal contacts (Porras-Segovia p287)  "I had to learn better ways of coping than acting out against myself or others in anger, impatience or frustration (Chesin p.1351)  "mindfulness was never my strong suit... now I am more well-versed in it and aware of warning signs" (Chesin p.1351) |
|  | Providing motivation to change | Reasons for living | Participant quote: "I think perhaps these events made me open to something different, that the time and mind-set was right. I knew I could feel better... knowing that I could feel 'normal', I was desperate - and motivated - to try anything with potential (Chesin, p.1351).  "A desire to have support to improve their own mental health" (O’Connor p.9).  Interpersonal connections in safety plans were woven into reasons for living (e.g. seeing X grow up"; 'travelling and seeing my interstate friends'; 'a cottage in Ireland or Scotland with X' and coping strategies, which frequently referred to being in the presence of other people (e.g. 'spend time at X's', 'watch X [child's name]; 'go on a drive with parents', or reflecting on relationships e.g. 'read letters to me from important people', 'think about my loved ones', X, our future together' (Rainbow (1) p.367)  ‘I was at the point where I was willing to take any help given to me’ (O’Connor, p.9) |
|  |  | Therapeutic Relationship: facilitated reflection | "Once I spoke to [clinician] and that I felt comfy, at ease, it was easy... somebody that you can sit and talk to and get on with, that understands (O’Connor p.9)  The intervention works by creating a space for patients to express what is important to them; facilitating insight and reflection, validating emotions and building self-esteem, helping patients manage boundaries and trial what works to support their mental health (Shah p.6)  The intervention reimagines the practitioner-patient relationship and facilitates trust and new possibilities for patients who have difficult relationship and help-seeking histories (Shah p.7)  Inspire patients and increase investment in the intervention 'it's like I see that someone really can help me, so I open up and I try to do everything, you know, positive, to find anything positive you know, and go that way. So it's like you know that you know, the professional wants to help you, so you do everything you know, to help yourself first (Shah p.7) |
|  | Group norms | Peer-to-peer | Many reported that the ability to connect with other veterans having similar experiences and receiving input from others helped them feel supported. Many reported that they felt that fellow Veterans were like brothers and sisters who heal together. Additionally, PLF-T created a safe space to open up to others, to connect through facial expressions, and to give and receive words of support from group members (Patel p.277).  "Well we are Veterans and we are brothers and sisters. So, we wanna heal because you might have something that, that I have and you might have some treatment that make him feel better. We help each other out" (Patel P.277).  "And um just you know having other people in this group with similar situations gave us an opportunity to brainstorm um ways to deal with it better, you feel me. Just having different people's ideas and mind working at the same time. I thought that was a really great" (Patel P.277).  "I mean, let's face it like, you know, bringing up a conversation about how I feel suicidal is not, is not something you would do at dinner with your friends. So just being in a group with people who were talking about it and sharing those thoughts and those experiences they were having, I mean I just opened up right away. For me it was just good right away, it was just, it was an instant connection to the telehealth group (Patel P.277). |
|  |  | Group Dynamics | Inclusive and equitable participation. Many participants commented that group facilitators tried to ensure equitable participation and create space for each group member to speak even though they were not in person. Participants attributed this to the facilitators' ability to invite all group members via telehealth to participate, even those on the phone (Patel p.275).  "Very pleasant, very amicable, very sociable. I guess, inclusive, always felt like they were always rotating properly as far as getting enough feedback and, or enough involvement from each veteran, equally distributed throughout the session. So I really appreciate that about the instructors (Patel P.275). |
|  | Increasing self-awareness | Increasing self-knowledge, insight and self-management skills | "This is really working for me. I'd been in talk therapy and on prescription medication for 15+ years and was still in a bad place. (Chesin p.1351)  "I feel it has helped me open my mind to allow myself to sit with difficult emotions and not act on urges immediately" (Chesin p.1351)  Deterrents stopping participants from wanting to die or act on suicidal thoughts significantly increased (Goodman, 2022 p698).  A total of 47.4% of participants who created a safety plan made changes during the follow-up call phase (O’Connor p.10).  Two most commonly changed steps were steps 2 ("Things I can do to take my mind off my problems without contacting another person") n=19, and step 4 ("People in their personal lives to contact for support") n=18. (OConnor p.9)  Longer-term impact was reported by participants (e.g improved warning sign awareness) (O’Connor p.9)  Suicide-related coping was strongly positively associated with perceived usefulness (Rainbow p.287)  Cognitive reframing and distraction: participants who listed warning signs characterised by internal narratives 'e.g. negative self-talk, suicidal or self-harm ideation and hopelessness) appeared to recognise a subsequent need to occupy their minds. (Rainbow (1) p370)  One participant who had a particularly extensive list of activities named difficulty with idea generation itself as a warning sign 'not knowing many things that would occupy me or be enjoyable', underlining their reasons for preparing a comprehensive distraction strategy (Rainbow (1) p.370).  'Planning for the future remind me to think twice about the thoughts going on my head' (Rainbow (1) p.370).  Emotional regulation: for some, recognition of a suicide crisis involved an emotional-sensory overlap with warning signs characterised by feelings within the body 'increased sensitivity to sound, light and movement', 'clenched jaw', 'I'm in physical pain' and recognition of related, negative emotions 'short tempered', 'crying at the drop of a hat', 'becoming bitter' (Rainbow (1) p.370).  "As group sessions progressed, I became more aware of triggers from working with the safety plan, and I was pleased to be able to complete the mindfulness exercises" (Chesin p.1351) |
| Changes related to behaviour: how and why individuals modify their habits and actions to achieve goals and improve their wellbeing | Countering Suicidality | Employing strategies to counter warning sign(s) & trigger(s) | For some participants, resolving disconnection by reaching out appeared in 'make my space safe' entries (e.g. 'stay in the company of others'; 'be in common areas of the house', 'if X is home, tell him I'm experiencing an orange or red day so he can help with making the space safe' (Rainbow (1) p.367)  Direct countering of negative thoughts with positive ones in 'reasons for living' (e.g. 'thinking of the hard work I've done so far and the joy it brings me'; 'I have dignity and worth because God gives me dignity and worth' (Rainbow (1) p.370)  Coping strategies responding to disruptions to regular routine (warning sign), countering this by focusing on maintaining routine and practicing self-care ('work in the garden' This suggests that for some participants, disruptions to routine are seen as the start of a spiral that can be arrested by consciously maintaining productivity (Rainbow (1) p.370) |
|  |  | Employing strategies to match or neutralise warning sign(s) & trigger(s) | 'I feel like a burden [warning sign], it's best to be alone [coping strategy]' (Rainbow (1) p.367)  ''I feel like I don't belong' [warning signs], 'get in the car and park somewhere quiet to ride out the storm [make my space safe] (Rainbow (1) p367).  For some people suicidal distress leads them to choose solitude over connection (Rainbow (1) p.367)  Many participants choosing coping strategies that appeared designed to match or neutralize the emergence of specific warning signs, such as changes in thought or sensory patterns, disruptions to routine or lapses in self-care (Rainbow (1) p370).  Responding to disruptions to regular routine (warning sign) countering this by taking the day off. For these participants disruptions are seen as a warning sign of overwhelm that indicates self-care is needed (Rainbow (1) p.370) |
|  |  | Employing strategies to counter emotional overwhelm | Countering emotional overwhelm by using mindful activities focused on the body 'I start to dissociate [warning sign] 'stretching, weights, dance or walking' [things I can do by myself] (Rainbow (1) p.370).  Countering emotional overwhelm by doing self-care activities that engaged senses such as taste 'bake something sweet', smell 'use essential oil diffuser', touch, 'weighted blanket' or a combination 'walk to the shops for chocolate milk', 'using sensory box'. (Rainbow (1) p.370)  Countering emotional overwhelm by modifying their environment/space 'make my room feel calm - light a candle, put on diffuser, turn on mood lighting' OR deliberately seeking out a change of environment 'e.g. got to movies, even alone', 'go for a walk by myself to clear my head' (Rainbow (1) p.370).  Using technology to shift the mood state e.g. YouTube / going on phone / look things up on my phone / watch stand-up comedy shows (Rainbow (1) p.370)  "The practices [mindfulness] do give me a little space to escape from the feeling that I'm a puppet being yanked hither and thither by my emotions" (Chesin p.1351) |
|  |  | Countering death wish with reasons to live | 'my little sister so she doesn't have to deal with stuff alone', 'seeing my kids grow up' (Rainbow (1) p.387). |
|  |  | Interactive Distraction | Direct countering of suicidality by displacing distressing thoughts in solo coping strategies through distraction e.g., 'watching movies, good or bad', 'listen to loud music' (Rainbow (1) p.370)  Direct countering of suicidality by doing cognitively absorbing or creative activities (e.g. 'doing puzzles', 'paint or crotchet', 'woodworking' (Rainbow (1) p.370)  Using technology e.g. ‘play sims’, play video games (Rainbow (1) p.370)  Using friends and family face-to-face social connections were preferred, some preferred to list technology enabled virtual connections ‘text a friend an interesting question’ or use online peer support forums / join an online community (Rainbow (1) p.370) |
|  |  | Maintaining wellbeing | "I will continue with yoga and a meditation group" (Chesin p.1351).  "Now mindfulness practices are a daily part of my routine, as well as something I rely on to ground me in moments of discomfort" (Chesin p.1351) |
