# Supplementary material for: An Empirically Based, Recovery‐Orientated Framework for Safety Planning Interventions for Suicide Prevention: An Integrative Literature Review and Critical Meta‐Autoethnographic Synthesis
Source: J Psychiatr Ment Health Nurs. 2026 Sep 9;33(6):1060–81. doi: 10.1111/jpm.70202 (PMC13748692; doi:10.1111/jpm.70202)
Supplement: Supplementary file 3 [file JPM-33-1060-s003.docx]

| **Conceptual Framework for Personal Recovery** | **Example quote(s) in the literature** |
| --- | --- |
| Connectedness | ‘Just having different people’s ideas and minds working at the same time. I thought that was really great’ (Goodman p.14 & Patel, p.277)  ‘when somebody point something out to you and then you suddenly began noticing it all the time when you hadn’t thought about that before’ (Shah p.6).  ‘We help each other out’ (Patel, p.277)  ‘sometimes you’re just so caught up in what you’re thinking of you can’t think anything bigger until someone kind of questions you a little bit so that then you end up questioning yourself, and you’re able to do these things’ (Shah, p.6).  ‘we wanna heal because you might have something that, that I have and you might have some treatment that make him feel better’ (Goodman p.10 & Patel, p.277).  ‘Many reported that the ability to connect with other Veterans having similar experiences and receiving in put from others helped them feel supported’ (Goodman, p.10) |
| Hope & Optimism | ‘The intervention mostly gives agency and hope to highly distressed patients’ (Shah, p.05).  Participant quotes: ‘what if tomorrow goes better?’ / ‘I want to discover all the good things that have to come’ / ‘someday I will be big, not famous or a millionaire, but someone truly happy’ / ‘I want to finish my degree and become a nurse because it’s my dream’ (Porras-Segovia p.289).    Participant quote: ‘I was at the point where I was willing to take any help given to me’ (O’Connor, p.9)  Participant quote: ‘I think perhaps these events made me open to something different, that the time and mind-set was right’ (Chesin, p.1351). |
| Meaning & Purpose | Safety plan entries ‘I have dignity and worth because God gives me dignity and worth’ / ‘my little sister so she doesn’t have to deal with stuff alone’ (Rainbow (1) p.369-370) ‘seeing my kids grow up’ (Rainbow (1) p.389). |
| Empowerment | ‘the practices do give me a little space to escape from the feeling that I’m a puppet being yanked hither and thither by my emotions’ (Chesin p.1351).  ‘[the intervention] helped me to connect the dots a little better and to come up with um a game plan on how to um deal with things, cope with things a little better’ (Patel, p.278).  Enabling patients to trial out different strategies within a safe space and review with practitioner. ‘so it was almost like we try Trial-and-errored parts of the safety plan to see how it worked in practice as opposed to just in theory’ (Shah p.7) |

**Supplementary File S4. Personal recovery results**

n.b. many of the concepts of recovery could equally apply to the literature – this is a selection of quotes.
