# Supplementary material for: An Empirically Based, Recovery‐Orientated Framework for Safety Planning Interventions for Suicide Prevention: An Integrative Literature Review and Critical Meta‐Autoethnographic Synthesis
Source: J Psychiatr Ment Health Nurs. 2026 Sep 9;33(6):1060–81. doi: 10.1111/jpm.70202 (PMC13748692; doi:10.1111/jpm.70202)
Supplement: Supplementary file 4 [file JPM-33-1060-s004.docx]

**Supplementary file S2. Completed TIDieR Checklist included studies**

| **Lead Author / Date** | **Item Category** | **Description** |
| --- | --- | --- |
| Boudreaux, 2017 | Brief Name | Web-based, self-administered safety planning application (p.1) |
|  | Why | Increase time efficiency of building a safety plan according to the Safety Planning Intervention, to help individuals in managing suicidal thoughts (p.2) |
|  | What: Materials | Web-based self-administered safety planning application that could be completed on a desktop, laptop or tablet computer. Designed to be used as a clinical tool in healthcare settings. App included standardized instructions, “as needed” access to video instructions, and ready downstream access to the safety plan for Web-based editing, reprinting, and sharing with other clinicians and caregivers. Rapid, user-friendly completion, multiple choice options and a free-text included for each step to allow individuals to input their own responses. Each of the six traditional SPI steps is presented sequentially, with easy-to-follow instructions and a “MORE” button that provides additional detail, if needed. A brief instructional video was developed to provide additional instructions to the patient, accessible as needed (p3) |
|  | What: Procedures | Once all six steps completed, the individual reviews and “confirms” the safety plan. The printed version is formatted similar to the traditional SPI and is designed to fit on a single page for ease of printing, access, and manipulation. The SP is stored on a secure server, and the individual can access the plan from any computer or mobile phone with Internet connection. The SP can be edited, printed, saved as a portable document format (PDF) and shared with others by email through secure email service (p3.) |
|  | Who: Provided | Not intended to replace the clinician but to make building safety plans more efficient for the clinician by reducing the total time required to build a safety plan by offloading the bulk of the orientation of the safety plan and the creation of the plan itself to the computer. The clinician will still maintain a role in reviewing the safety plan and editing it, as needed. (p.2). 40% participants reported or demonstrated at least one problem related to computer literacy, including unfamiliarity with using the mousepad and with typing on a keyboard. Required technical assistance. (p5). 23% reported or demonstrated problems with the actual process of building the SP. Initial registration process caused some confusion (strong password). (p5). Some needed clarification on how to complete individual steps (p.5). |
|  | How | Individual delivery through computer application; assisted face-to-face by research assistant (p.3) |
|  | Where | US urban, tertiary care hospital; emergency department or inpatient psychiatric unit (p.3) |
|  | When and how much | Single session. (p.3) |
|  | Tailoring | Multiple choice options were developed for the first three steps. A free text field was included for each step to allow individuals to input their own responses, if and when they desire. (p.3) |
|  | Modifications | Combination of multiple choice and free-text balanced the need for simplicity with the goal off personally tailored planning. Each of the six traditional SPI steps is presented sequentially, with easy-to-follow instructions and a “MORE” button that provides additional detail, if needed. A brief instructional video was developed to provide additional instructions to the patients, accessible as needed (p.3) |
|  | How well: planned | To remove the limitation of being dependent on a patient having a mobile phone, downloading the app successfully and completing the safety planning steps, most of which require extensive text entry, during a clinical encounter, review by the clinician on a patient’s phone is not practical. We designed, built and tested the usability of a Web-based, self-administered safety planning application that could be completed on a desktop, laptop, or tablet computer. Our goal was to have the application include standardized instructions, “as needed” access to video instructions, and ready downstream access to the safety plan for Web-based editing, reprinting, and sharing with other clinicians and caregivers. We do not intend for the Web-based application to replace the clinician; rather, we expect it will make building safety plans more efficient for the clinician by reducing the total time required to build a safety plan by offloading the bulk of the orientation to the safety plan and the creation of the plan itself to the computer. The clinician will still maintain a role in reviewing the safety plan and editing it, as needed (p.2) |
|  | How well: Actual | All participants successfully registered, viewed all six of the safety planning build screens, and created a final, one-page safety plan PDF that could be printed, saved, or shared by secure email. However, completeness of the safety plan varied. Some participants experienced some type of technical failure, including interrupted Internet connection and “frozen” screen resulting from a glitch in the Morae usability software. 40% participants reported or demonstrated at least one problem related to computer literacy, including unfamiliarity with using the mousepad and with typing on a keyboard. This required some technical assistance from RA. Other issues included, initial registration process caused confusion, participants needed clarification on how to complete individual steps. Interruptions during the process were relatively common due to testing, meals and visitors. Only 17% chose to watch instruction video. (p5-6) |
|  | | |
| Chesin (2015) | Brief Name | Mindfulness-based cognitive therapy to prevent suicidal behaviour (MBCT-S) (p.1345) |
|  | Why | To reduce suicide attempt risk by increasing awareness of individual warning signs of suicidal crisis, suicide-related coping skills and personal and professional contacts who may aide in increasing immediate safety. These goals are targeted most directly during safety planning. Longer-term goals of MBCT-S are to train attentional control so that individuals may stay out of intractable suicidal and hopeless thoughts that lead to suicidal crisis in the first place. (p.1348) |
|  | What: Materials | SPI – six steps of SPI (Stanley & Brown 2012) – completion of written safety plan that lists suicide coping strategies, social contacts who may serve to distract from suicidal thoughts, family members or friends to call for help, and MH professionals or agencies to contact in suicidal crisis or when rescue is needed. A plan for limited access to lethal means is also made and documented on the safety plan, and warning signs of impending suicidal crisis are identified and included on the plan so that the individual may recognise when use of his or her safety plan is indicated. MBCT – S: combine cognitive therapy exercises with mindfulness-meditation practices and discussions of participant experiences during therapy exercises. Suicide-specific content includes systematic review and rehearsal of the safety plan throughout treatment, didactics on risk factors and symptoms associated with acute suicide risk and longer-term suicide attempt vulnerability, psychoeducation on the cognitive model of suicidal behaviours, and group discussion of suicide coping skills (which may include distraction techniques and grounding or other brief mindfulness-meditation practices). Suicide risk assessment are conducted at the beginning of every MBCT-S treatment session. Home mindfulness-meditation practice is assigned |
|  | What: Procedures | Participants first receive SPI individually followed by 8 weekly group MBCT-based treatment. Home mindfulness-meditation practice is assigned (3 days). Participants are asked to review their safety plan daily and to monitor their suicidal thoughts and urges on a daily basis using a worksheet designed for the MBCT-S. (p.1346) |
|  | Who Provided | Lead author (MC) delivered all MBCT-S sessions to all groups. Prior to providing MBCT-S she had 5 years of experience providing safety planning to suicidal patients and mind-body interventions to community member participants. She had also received advanced training in SPI and MBCT. She is one of the MBCT-S treatment developers. (p.1348) |
|  | How | A nine session treatment. Session one is provided individually. Sessions two through nine are delivered in a group format and provided weekly. (p.1348) |
|  | Where | US Psychiatric outpatient clinic (p.1345) |
|  | When and how much | SPI – very brief (20-45 min) followed by Eight sessions of weekly group MBCT-based treatment follow (2 hr) (p.1346) |
|  | Tailoring | Time lags between the pretreatment assessment and the first treatment session were due to the logistical difficulties involved in forming a cohort of sufficient size.(p.1348) |
|  | Modifications | ? |
|  | How well: planned | Provider participated in MBCT training and weekly consults with another MBCT-S developer; number of sessions attended by participants recorded; participants self-reported time spent on home practice in last 4 weeks (p.1348-1349) |
|  | How well: actual | Participants on average attended 7 sessions (range: 5 to 9), spent on average 30 minutes per day, 5 days per week on meditation and 9 minutes per day, 2 days per week reviewing their safety plan (p.1349) |
|  | | |
| Goodman, (2022) | Brief Name | Dyadic safety planning intervention (Safe Actions for Families to Encourage Recovery (SAFER)) (p.1) |
|  | Why | To involve family members in the Safety Planning Intervention for additional support/feeling of belongingness, to encourage treatment adherence, notice and cope with suicidal symptoms, improve communication (p.4) |
|  | What: Materials | intervention manual with session protocols for providers (not accessible in study) (p.4) Safety plan: Veterans: step 1 warning signs; step 2: internal coping strategies; step 3: people and social settings that provide distraction; step 4: people whom I can ask for help; step 5: Professionals and agencies to contact for help; step 6: Making the environment safe. Safety plan Supporting partner: step 1: recognizing warning signs/raising with Veteran; step 2: coaching Veteran on use of coping strategies; step 3: facilitating Veterans’ use of supportive social contacts; step 4: Providing direct support; step 5: Facilitating contact with professionals/agencies; Step 6: Making the environment safe. Session content:  Joining sessions – held separately |
|  | What: Procedures | Developed as a dyad-based treatment to help Veterans cope more effectively with their MH symptoms, improve communication, decrease feelings of burdensomeness, and increase social support (e.g. sense of belonging). Targeted family members by providing them with tools to help Veterans cope, improve their communication with one another, and decrease a family members’ own feelings of burden. (p.44) |
|  | Who Provided | Psychiatrist, psychologist and mental health counsellor, who each had extensive training in either cognitive behaviour or dialectical behaviour therapies, and suicide risk assessment. All attend 1-day training to review treatment manual components, instruction in dyad therapy principles, and suicide risk management. (p.45) |
|  | How | Joining session held separately with Veteran and supporting partner. Subsequent sessions group (p.45) |
|  | Where | Face-to-face outpatient clinic |
|  | When and how much | 90-min, four session. First three sessions conducted in consecutive weeks with the last session serving as a booster (3 weeks after the third session). The entire treatment completed within a 6-week period (p.44) |
|  | Tailoring | ? |
|  | Modifications | ? |
|  | How well: planned | The interventionists followed the manual and received supervision after sessions by treatment developer (p.48) self-developed fidelity scale with 4 session-specific, 13 general clinical competence items, all sessions video-recorded, 7 sessions randomly selected and rated by one trained rater; providers below 80% rating received additional supervision and adherence monitoring |
|  | How well: actual | average ratings of providers ranged from 82 to 100%; mean score 90.7; no drops below 80% threshold |
|  | | |
| Goodman, (2021) & Patel (2023) (qualitative study) | Brief Name | Project Life Force (PLF) (p.691) |
|  | Why | To enhance the meaningfulness and personalisation of the safety plan with the necessary guidance in emotional regulation, interpersonal/friendship and distress tolerance skills (p.691) |
|  | What: Materials | Teaches skills that target: 1) aggression and impulsivity; 2) medication and treatment adherence; 3) emotion regulation strategies to alleviate psychological distress and hopelessness; 4) reducing access to means; 5) asking for support and help. PLF augmented with modules about strengthening friendships, education pertaining to suicide risk, suicide means reduction, and suicide prevention mobile application. Alongside completing Safety plan.  Session 1: introduction, psychoeducation about suicide, safety plan step 5: crisis numbers, meet local suicide prevention coordinators. Session 2: safety plan step 1 – identification of warning signs; session 3 – safety plan step 2 – internal coping strategies; session 4 SP step 3 – identifying people to help distract; session 5 SP step 4 – sharing safety plan with family; session 6 safety plan step 5 – professional contacts & step 6 making the environment safe; session 7: improving access to the safety plan; session 8 physical health management; session 9 building a positive life; session 10 recap/review; (p.692) |
|  | What: Procedures | Each PLF session’s format includes: (1) a brief check in and follow up pertaining to use of the safety plan, (2) homework review, (3) teaching of new material and skill, (4) in-class practice of the skill, including role plays, and (5) assignment of homework/outside practice/development of the safety plan. PLF has an open-group format, with new members joining at any session throughout the 10-week cycle. This permits members to join at any time, allowing for immediate access to the intervention to accommodate the need for expeditious safety planning for these higher risk Veterans. As the PLF program runs continuously, Veterans who join after session one, can cover missed material in the next cycle. |
|  | Who Provided | Clinician |
|  | How | Group format led by clinician. Face-to-face |
|  | Where | Outpatient clinic |
|  | When and how much | 90-mins psychotherapeutic group treatment lasting 10-weeks.. Clinician time: an average of 2 hours a week per PLF clinician required totalling four hours per session. (p.697) Patel study: virtual, online group delivery |
|  | Tailoring | Open group format enables individuals to enter the group immediately upon discharge as have the ability to pick up misses sessions in the next round (p.692) Patel: adaptation to telehealth delivery by group facilitator and project coordinator (p.3) |
|  | Modifications | Patel: using a communications coordinator for outreach and engagement, providing telehealth orientation session, restructuring sessions, emailing and screen sharing the intervention manual and safety plans (p.3) |
|  | How well: planned | Session 11 add on session if required – dealing with a suicide death of a group member. (p.692) |
|  | How well: actual | The open-group format provided maximum flexibility, facilitating hospitalized individuals to enter the group immediately upon discharge as well as ability to pick up missed sessions in the next round. As a consequence of this format, some members reviewed the steps of the safety plan out of order, but this was not a concern for our group participants, who all entered the group with an initial safety plan that was enhanced during the PLF  program. Additionally, with an open-group format, developing group cohesion can be more challenging when there are multiple revisions to group membership in a short period of time. However, in most sessions the majority of group members had attended previous sessions so there was continuity of the group process. The beginning of each session is dedicated to introductions of new members, such that each Veteran knows  the name and background of the other group members. Veterans who held seniority by having attended more PLF group sessions at times served as role models for newer members, who were more symptomatic. (p.699) Patel: 68% of participants attended 7 or more sessions (p.4) |
|  | | |
| Melvin (2018) | Brief Name | Beyond Now safety planning smartphone application |
|  | Why | Provides a platform for individuals to create, edit, access, and share their personalized safety plan (p.848) |
|  | What: Materials | Safety planning smartphone application (app). Based on the SPI (Stanley & Brown, 2012). Users are able to list warning signs, reasons to live, ways to limit access to lethal means, coping strategies, and personal and professional contacts. Also includes a red emergency phone button on each page, which allows quick access to preloaded Australian emergency service phone numbers, and also has an information section containing a video outlining the practice of safety planning as well as a link to further information. (p.848) |
|  | What: Procedures | Once a safety plan has been created, it can be viewed easily in a single scroll-down page, edited, or shared via email. (p.848) |
|  | Who Provided | Self-administered |
|  | How | Through users’ compatible smartphone |
|  | Where | Anywhere |
|  | When and how much | Majority of participants reported using the app “occasionally” (63.6%) or “a lot” (13.6%) as opposed to “not at all” (0%) or “rarely” (22.7%) (p.852). |
|  | Tailoring | Information is entered into the app using free text or by selecting from a drop-down list of suggestions. Personal and professional contact can be directly imported from the user’s phone directory. |
|  | Modifications | n/a |
|  | How well: planned | n/a |
|  | How well: actual | n/a |
|  | | |
| O’Connor (2022) | Brief Name | SAFETEL safety planning and telephone follow-up intervention |
|  | Why | To reduce suicidal behaviour (p.1) |
|  | What: Materials | Pen and paper completion of SPI six steps involving person-centred warning signs and helpful responses to an imminent suicidal crisis Plus follow-up telephone calls post discharge (p.3) |
|  | What: Procedures | During follow-up calls, patients’ mood and current level of suicidality explored, and appropriate escalation procedures followed if found to be at imminent risk of suicide. Use of the safety plan and engagement with any supports (personal and professional), since last contact, was explored to help facilitate engagement with both the intervention and other services. People also given the opportunity of updating their safety plan. (p.4) |
|  | Who Provided | Emergency department clinicians, NHS Liaison psychiatry team (p.3) Collaborative emergency safety plan developed by the patient in collaboration with a trained practitioner. |
|  | How | In-person and then follow-up by telephone. (p.3) |
|  | Where | Initially in emergency department and then after discharge by telephone (p.3) |
|  | When and how much | Up to five optional telephone calls (the first call within 72 h of patient discharge from hospital and then weekly thereafter depending on patients’ availability). (p.3) |
|  | Tailoring | ? |
|  | Modifications | Follow-up call engagement was recorded for each intervention participant (p.6) |
|  | How well: planned | Audio recording of SPI sessions, randomly selected sample (30% in the intervention phase) assessed independently by two intervention researchers, using the SPI rating scale (p5) |
|  | How well: actual | Average fidelity score of audio ratings of 91% in the piloting phase, 84% in the intervention phase, >68% of evaluated recordings completed over 80% of the SPI (p.8) |
|  | | |
| Parrish (2023) | Brief Name | Safety and Recovery Therapy (mSTART) (p.1640) |
|  | Why | To increase engagement with the Safety Planning Intervention and implementation of a suicide safety plan among people with SMI, through a CBT intervention based on the Safety Planning Intervention, mobile augmentation to increase knowledge and awareness of the safety plan, telephone follow-up calls to connect participants to outpatient care (p.1641) |
|  | What: Materials | Therapy designed to improve self-efficacy in coping with suicidal symptoms by implementing crisis-management strategies (SPI). Reviewed early warning signs and triggers for a suicidal crisis, as well as coping strategies for these triggers. A Safety plan that also included a list of people they could call for distraction and/or help in a crisis, crisis lines, and lethal means counselling. Mobile augmentation of therapy content including general CBT techniques, current mood, and queries about safety plan elements. Relevant to safety planning, individuals were queried once a day via mobile phone if they noticed any of their early warning signs, and if so, whether they engaged in a skill or coping strategy (e.g. thought challenge, behavioural coping strategy) derived in person meetings. (p.1640) |
|  | What: Procedures | An open-ended safety planning approach. Follow-up phone contacts. (P.1641) |
|  | Who Provided | Therapist and clinician for follow-up phone contact (p.1641) |
|  | How | Therapy in-person and telephone calls |
|  | Where | Psychiatric walk-in clinics and community mental health centers |
|  | When and how much | Four sessions, once a week (with therapist). Telephone contacts at weeks 6,8 and 10 following treatment or until they connected with outpatient care. Mobile augmentation of therapy content for 12 weeks following the intervention (p.1641). |
|  | Tailoring | The number of strategies each individual discussed with their therapist is not standardized. Mobile augmentation was personalised to individuals’ unique safety plan (p.1641). |
|  | Modifications | ? (secondary analysis study) |
|  | How well: planned | n/a |
|  | How well: actual | n/a |
|  | | |
| Porras-Segovia (2024) | Brief Name | MEmind Smartphone app (p.285) |
|  | Why | Digitalise suicide safety plans based on SPI to increase availability and personalisation (p.285) |
|  | What: Materials | Digital safety plan embedded in the Memind app, free, compatible with iOS and Android, and requires a username and password. Safety plan is composed of seven tabs corresponding to the seven parts of the original safety plan. 1. Warning signs – signs that alert patients of an incoming crisis. 2. Internal coping strategies – suggestions for distracting activities that patients can do on their own without recourse to the external environment. 3. External coping strategies – these are suggestions for distracting activities that patients can do outside the home or with the support of the social environment. 4. Personal contacts – personal contacts that make up the patients’ support network. 5. Professional contacts – contacts of professionals or institutions to contact in a crisis situation. This may include telephone numbers, locations or links to mapping applications. 6. Safe environment – recommendations for making the usual environment free of lethal means. 7. Reasons to live – these are the most important reasons that may motivate patients to stay alive. As this is a digital plan, each of these tabs, in addition to adding text entries, can include images, videos, links to websites, audios etc. (p.285 - 6) |
|  | What: Procedures | Username and password created by clinician so it can only be used by patients and study participants. Users can access the security plan from the main menu on the app’s home screen to set it up and activate it. During the first interview with the referring psychiatrist, patients make a first configuration of their safety plan. |
|  | Who Provided | Clinicians in emergency department, in medical units, in the mental health inpatient unit and in the mental health consultations of two hospitals. (p.286) |
|  | How | Patients who owned a smartphone with internet access and iOS or Android operating system. And if institutionalised or incarcerated had access to regular mobile phone use. (p285) |
|  | Where | Digital, mobile phone use – anywhere |
|  | When and how much | Once to set up initial safety plan with clinician and subsequently as often as users wished (p.285) |
|  | Tailoring | Safety plan can be customised as many times as users wish (p.285) |
|  | Modifications | Modifications suggested in study by patients, but none during study. |
|  | How well: planned | Participant survey to record number of times the safety plan was used with or without the app (p.287) |
|  | How well: actual | Mean number of SP activations of 9 times over mean of 285 days of follow-up (287). |
|  |  |  |
| Rainbow (2024) & Rainbow (1) (2024) | Brief Name | Beyond Now safety planning smartphone app |
|  | Why | To reduce suicidal ideation and increase suicide-related coping by using a digital smartphone safety planning app (p.295). To provide a platform to create, edit, access, and share a personalised suicide safety plan based on the SPI to reduce suicide risk (1) – p.364). |
|  | What: Materials | Mobile application, freely available to download in Australia on both iOS and Android platforms from Beyond Bule website. Designed specifically to enable users to create a personalised safety plan to support them through a suicide crisis via prioritized steps based on the structure of the SPI (Stanley & Brown, 2012). Step 1. My warning signs; 2. Make my space safe (means restriction); 3. My reasons to live; 4. Things I can do by myself (internal coping strategies); 5. People and places I can connect with (for distraction); 6. People I can talk or yarn to (for disclosure); 7. Professional support. (p.364-365) |
|  | What: Procedures | Users can enter free text for each step, or choose from a number of in-app suggestions (e.g. I feel like a burden) in warning signs, ‘things I haven’t done yet’ in my reasons to live). An option to upload photos and videos is available for steps 3 through 6. Plan content is stored only on the user’s phone but can be shared via email or SMS if the user chooses to do so. (p.365) |
|  | Who Provided | Users themselves / self selected (p.365) |
|  | How | Downloaded the app via the website (p.365) |
|  | Where | Users own mobile phone |
|  | When and how much | When they wanted to, as often they wished |
|  | Tailoring | Overall plans demonstrated a high level of personalisation with respect to original rather than app-generated content (p.367) |
|  | Modifications | None |
|  | How well: planned | N/a |
|  | How well: actual | n/a |
|  | | |
| Shah (2024) | Brief Name | The Assured Intervention (p.3) |
|  | Why | To reduce repeat self-harm and/or suicidal ideation presentations to hospital and improve experiences of care and mental health (p.3) |
|  | What: Materials | Enhanced biopsychosocial assessment and rapid follow-up care. Enhanced biopsychosocial assessment consists of two components: narrative interview and safety plan. (p.3) Narrative Interview. Safety plan SP identified person’s warning signs, internal and external coping strategies, informal and formal support to improve awareness and self-management of future self-harm (p.4). Follow up care: check-in phone call. Follow-up sessions: practitioner worked with the person to explore their future hopes and then identify the possible resources and strengths already present in achieving or working towards their future hopes (p.4) Letters: from the practitioner to remind them of the safety plan and support networks (p.4) |
|  | What: Procedures | Practitioner began the biopsychosocial assessment with a narrative interview that invited the person to tell their story leading up to the crisis (p.4) Use open questions skilfully and consistently to ensure the patient decided the content that was discussed. The narrative interview also sought to develop a therapeutic alliance to engage patients and to help them feel hopeful and supported. Use of active listening skills, validation, and hope-instilling statements was covered in the training. Once practitioner heard the person’s story, they then conducted their biopsychosocial assessment. (p.4) At end of biopsychosocial assessment the practitioner worked with the person to develop a personalised and enhanced safety plan based on SPI (Stanley & Brown, 2012). Use open questions with the aim of empowering the patient to draw upon their own expertise and skills to complete safety plan, rather than the practitioners. This safety plan differed from the typical safety plan carried out in the practitioner usual role in that practitioners worked with the person to identify barriers to using these strategies, and subsequently steps to overcome said barriers, to maximise the potential for them using this in future crises. (p.4). Rapid follow-up care after discharge from hospital. The person received a check-in phone call within 72 hours of leaving the emergency department from the same practitioner (p.4). Follow-up sessions with the same practitioner using a solution-focused approach. (p.4) The person receives a personalised letter from the practitioner to remind them of the safety plan and support networks (p.4) |
|  | Who provided | Practitioners trained to carry out assessment with an emphasis on specific therapeutic principles and techniques to maximise the therapeutic potential of the assessment. (p.3) Training for the intervention entrusted practitioners to take a person-centred approach. (p.3) Safety plan coproduced (p.4) Training for follow-up sessions focused again on question techniques, such how to reframe questions in response to scenarios, and using scales to bring strengths and successes into the focus. (p.4) |
|  | How | In-person in Emergency department then via telephone and letter |
|  | Where | Emergency Department then post discharge |
|  | When and how much | Follow-up sessions over a two-month period at approximately 1, 4 and 8 weeks. Letters at three, six and nine months (p.4) |
|  | Tailoring | Person-centered and planned to be personalised and adapted to individuals’ preferences (p.5) |
|  | Modifications | For many practitioners solution-focused, person-centred, strengths based conversations required a shift from their usual approach which involves the practitioner directing the interaction with a focus on assessing risk, formulating needs, and outlining actions and a goals for the patient to follow (p.4) Mode of delivery after ED contact (online rather than in-person) chosen according to COVID pandemic situation, service and patient needs. (p.7) |
|  | How well: planned | Same practitioner would do all follow-up sessions and letters (p.7) |
|  | How well: actual | No letters sent to patients. Same practitioner struggled to keep continuity due to shift changes, leave etc (p.7) |
|  | | |
| Stanley (2018) | Brief Name | SPI + |
|  | Why | To enhance the effectiveness of SPI by combining it with structured telephone follow-up to reduce suicidal behaviour and enhance treatment engagement (p.895) |
|  | What: Materials | Original brief SPI: 6 steps = 1) identify personalised warning signs for an impending suicide crisis; 2) determine internal coping strategies that distract from suicidal thoughts and urges; 3) identify family and friends who are able to distract from suicidal thoughts and urges and social places that provide the opportunity for interaction; 4) identify individuals who can help provide support during a suicidal crisis; 5) list mental health professionals and urgent care services to contact during a suicidal crisis; 6) lethal means counselling for making the environment safer. SPI + adds a component that consists of telephone contact after discharge from the ED included 3 components: 1) brief risk assessment and mood check; 2) review and revision of the SPI, if needed; 3) facilitation of treatment engagement. |
|  | What: Procedures | Safety plans created with ED staff; follow-up contacts provided by social workers, psychologists (p.896) |
|  | Who provided | Project staff who were social workers or psychologists and trained and supervised by senior project staff (p.896) |
|  | How | 1-to-1 face-to-face delivery of SPI, telephone contacts follow-up (p.896) |
|  | Where | Emergency Department and then on telephone (p.896) |
|  | When and how much | Emergency Department having been admitted following a suicide related event. Telephone contacts were attempted within 72 hours of discharge. Follow-up outreach continued weekly and generally discontinued after at least 2 calls if the patient had at least 1 outpatient behavioural health appointment or no longer wished to be contacted (p.896). |
|  | Tailoring | number of follow-up phone calls dependent on participant preferences and/or scheduling of first behavioural health appointment (p.896). Safety plans intended to be personalised. |
|  | Modifications | Patients were able to opt out of the SPI+ during the ED visit or during follow-up calls (p.896) |
|  | How well: planned | Number of follow-up sessions attended by participants recorded (p.897) |
|  | How well: actual | 87% were able to be contacted at least once, participants completed a mean of 3.7 calls (p.897) |
|  | | |
| Vijayakumar (2017) | Brief Name | CASP: contact and safety planning intervention (p.589) |
|  | Why | To reduce suicidal behaviour among participants in refugee camps by providing emotional support, creating a safety planning card, and referring to professional services if needed (p.590). |
|  | What: Materials | Safety planning card consisted of an individualised list of coping strategies containing names and contact numbers of persons in the individual’s immediate family, social circle and health services who could be contacted during a suicidal crisis (p.590). |
|  | What: Procedures | Community volunteers provided emotional support to individuals who were suicidal or depressed. Each CV provided a list of individual identified to be at high risk. Each CV was allocated a fixed number of these individuals whom they visited twice a month. During the visit, emotional support was offered and suicide risk assessed. The CVs in consultation with each individual listed the warning signs, coping strategies and list of available support in the safety planning cards. Participants were encouraged to use the cards when in distress. After every visit, a short contact form was filled, which was based on the SUPRE MISS follow-up form. If there was an imminent risk for suicide ADRA and Sneha volunteers were immediately informed who then provided the necessary care and referrals. A monthly review of the contact forms and discussions with the CVs were undertaken by designated Sneha volunteers for clarifying doubts, initiating referrals and providing support to the CVs. Those who needed professional intervention were referred to the psychiatric department of the Government Hospital, (p.590) |
|  | Who provided | Community Volunteers (CVs) Underwent an intensive 20-hour training programme wherein communication skills, loss and grief, depression, suicide and empathetic offering of emotional support were communicated to them. They were also trained in obtaining informed consent and in filling the safety planning cards for each individual participant. |
|  | How | CVs worked in the refugee camps and met people there or talked to them at bus stop (p.591) |
|  | Where | In the refugee camps (p.590) |
|  | When and how much | Making periodic visits (p.590) |
|  | Tailoring | Personalised coping strategies and sources of support (p.590) |
|  | Modifications | Those who needed professional intervention were referred to the psychiatric department of the Government Hospital, but logistical and infrastructure difficulties proved a major deterrent (p.591) |
|  | How well: planned | Those who were deemed high suicide risk would be referred for psychiatric treatment (p.591) |
|  | How well: actual | After 4 months, the help of a private psychiatrist was sought who offered both his services and medication free of cost. Every month ADRA representatives escorted these patients for review (p.591). |
|  |  |  |
| Wilson (2022) | Brief Name | Peer-delivered suicide safety planning (p.1087) |
|  | Why | To offer peer-delivered SPI to increase meaningful engagement with safety planning, acceptability of safety planning, increase quality of safety plans, to reduce suicidal behaviour, reduce repeat hospital presentations (p.1087-1088) |
|  | What: Materials | SPI (Stanley & Brown, 2012 template. Includes identification of warning signs for possible impending suicidal crises, internal coping strategies, people and social settings that can provide distraction, people to ask for help when in crisis, professionals or agencies to contact during a crisis, and ways to make one’s environment safe. Individuals are also prompted to list one thing most important to them that is worth living for. (p.1089) |
|  | What: Procedures | Patients are required to provide written responses themselves. All completed safety plans were approved by mental health providers in the ED before being entered into the electronic medical record. Peers received biweekly feedback by the study team on the quality and completeness of the plans and adherence to study protocol. No revisions to safety plans were allowed during these feedback sessions. The peers also received a weekly debriefing by a licensed clinical counsellor throughout the study (P.1089). |
|  | Who provided | Peer Support Workers: Peers trained to only discuss safety planning with patients. Provider SPI training: all medical and mental health providers participated in the ED-SAFE (Emergency Department Safety Assessment and Follow-up Evaluation) which involved extensive training on the treatment of suicidal patients in the ED, including SPI delivery. Peer SPI training: “peer” referred to an individual who had experienced severe suicidal ideation or had survived a suicide attempt in the past and who was state certified as a peer recovery support specialist. Peers were provided approximately 12 hours of initial training. (p.1089) |
|  | How | Face-to-face (p.1089) |
|  | Where | In the Emergency Department (p.1089) |
|  | When and how much | A brief, one session intervention when someone had been admitted to ED following a suicide related event |
|  | Tailoring | Safety plan intended to be personalised (p.1089) |
|  | Modifications | Any additional amount of time needed to work with the patient again until safety plan was acceptable was added to the safety planning process (p.1089) |
|  | How well: planned | Peer safety plans had to be approved by mental health providers in the ED. If providers did not approve the safety plan, the peer was asked to work with the patient again until the plan was acceptable. (p.1089) |
|  | How well: actual | All peer-delivered safety plans created in the ED were approved by providers without revision (p.1090) Safety plans completed with peer’s guidance were more complete and of higher quality than those made with providers. Furthermore, although these plans took longer to create when patients worked with a peer instead of a provider, the increased time did not affect time to disposition or ED LOS. Additionally, peer-delivered safety planning was associated with reduced repeat visits to the ED in the subsequent 3 months (p.1091) |
|  | | |
